# Evaluation of NHS Injectable Medicines Guide users’ information needs related to the co-infusion of intravenous medicines: user survey and Delphi consensus study

**DOI:** 10.1136/bmjopen-2024-094211

**Published:** 2025-05-30

**Authors:** Virginia Aguado Lorenzo, Matthew D Jones

**Affiliations:** 1 Imperial College Healthcare NHS Trust, London, UK; 2 Pharmacy Department, Guy's & St Thomas’ NHS Foundation Trust, London, UK; 3 Department of Life Sciences, University of Bath, Bath, UK

**Keywords:** Delphi Technique, Drug Combinations, Nursing Care, Pharmacists, Information management

## Abstract

**Abstract:**

**Objectives:**

To explore intravenous drug compatibility information needs of National Health Service (NHS) Injectable Medicines Guide (‘Medusa’) users. To develop consensus expert recommendations on the intravenous drug compatibility information that Medusa should include.

**Design:**

A convergent parallel design. An online survey of Medusa users. A three-round Delphi project developed expert consensus.

**Setting:**

Community, secondary, tertiary and mental healthcare sites across the UK.

**Participants:**

142 Medusa users completed the online survey (114 nurses, 28 pharmacists). A panel of 16 nurses and 25 pharmacists currently practising in the NHS with professional expertise relevant to the compatibility of injectable medicines participated in the Delphi project.

**Primary and secondary outcome measures:**

Users’ ratings of the importance of different types of compatibility information. Consensus expert agreement on a six-point scale regarding the compatibility information that should be provided by Medusa.

**Results:**

97 (68%, 95% CI 60 to 75%) users were involved in the co-infusion of drugs at least weekly. Most users reported co-infusing intravenous fluids (n=116, 82%, 95% CI 75 to 87%), antibiotics (n=85, 60%, 95% CI 52 to 68%) and analgesics (n=81, 57%, 95% CI 49 to 65%) in the past year. They considered information on whether drugs are compatible/incompatible, and the concentrations and diluents in which this occurs, most important (Friedman test p<0.001, Dunn-Bonferroni pairwise comparisons p≤0.028). The expert panel also identified these topics as important for all infused drugs, along with information on time frames, supporting data and specific requirements for the intravenous line (median agreement rating of both professions ≥5 with IQR ≤1.75 in each case).

**Conclusion:**

The co-infusion of drugs is common, so supporting information continues to be important. The most relevant information is that identified by both users and the expert panel: whether drugs are compatible/incompatible, and the concentrations and diluents in which this occurs. The expert panel’s recommendation to provide information for all infused drugs contrasts with current more limited national recommendations.

**STRENGTHS AND LIMITATIONS OF THIS STUDY**

- Strengths include the consideration of the perspectives of both Medusa users and subject experts, and the comparison of the opinions of both nurses and pharmacists.
- In the Delphi project, the target sample size was achieved and responses to previously accepted statements demonstrate that group stability was achieved once consensus was reached.
- The sample size of the user survey did not achieve the target, but as variation in responses was smaller than anticipated, the sample size achieved still enabled a margin of error of 3.5–5.2%.
- Due to participation bias, the findings are likely to be more representative of clinicians with an interest in intravenous administration and a bias towards current information provision.
- More extensive pre-testing would provide greater assurance over the correct interpretation of the questions.

## Introduction

 Many critically ill patients receive numerous intravenous medicines, often given by continuous infusion. This means that often the number of concurrent intravenous medicines is greater than the number of venous access lines, resulting in the co-infusion of two or more drugs via the same lumen. This allows the co-infused drugs to mix in the intravenous line before dilution in the patient’s bloodstream. With certain combinations of drugs, this can result in incompatibility (the formation of insoluble solid particles in the infusion, which may or may not be visible) or instability (chemical degradation of one or more drugs).^1^ Incompatibility has been associated with pulmonary adverse effects, which have been fatal in infants,^2^ whereas instability will lead to reduced plasma concentration of the therapeutic drug(s) and potential adverse effects associated with degradation products. Professional standards therefore require that intravenous medicines are only co-infused if absolutely necessary, that compatibility is confirmed prior to administration and mixing occurs as close to the vascular access device as possible.^3^

Despite this, studies of intensive care units (ICUs) in Europe and North America have found that the co-infusion of intravenous infusions is common in both children and adults. A study in a paediatric ICU in Switzerland found 1447 drug combinations co-infused to 100 patients over 503 patient-days,^4^ and a study in 13 adult ICUs in Canada found co-infusion in 33% of patients.^5^ There is also evidence that a substantial minority (2–26%) of co-infused drug combinations are incompatible, although in many cases alternative administration schedules could have avoided this problem.^4^,^9^ There is therefore a clear need to provide clinicians with the information on intravenous drug compatibility needed to support their clinical decision-making while administering multiple medicines, and in the UK, this was recommended in National Patient Safety Agency (NPSA) Alert 20 for commonly used mixtures in specialist areas.^10^

Such information is available from a variety of textbooks, databases and charts, which vary in the amount of information provided, the number of drug combinations included and their usability.^11 12^ However, there has been very little research into the information required in the clinical environment to support the safe co-infusion of intravenous medicines. In the only published study, which reports focus groups with nurses from two ICUs in the Thames Valley region of England, participants relied on experience to confirm the compatibility of familiar drug combinations but used written resources for unfamiliar combinations.^13^ They preferred to use a simple compatibility chart, as it was readily available, quick and easy to use. However, it was limited by the number of drugs included, so other sources were also used. The nurses reported that the absence of compatibility data was the main challenge they faced and often led to workarounds, so they requested more comprehensive information on the compatibility chart, including pH.

To support the safe use of intravenous medicines, many hospitals in the UK provide clinicians with access to the National Health Service (NHS) Injectable Medicines Guide (‘Medusa’).^14^ This website provides simple lists of compatibility and incompatibility in a dedicated subsection of each drug guide. In line with the recommendation of NPSA Alert 20,^10^ this is limited by Medusa’s policies to drugs given by infusion that are routinely used in critical care and/or the emergency department (online supplemental appendix 1), but as this can be difficult to define, in practice, information is provided for most injectable medicines. The design (but not content) of the compatibility section was recently revised following a programme of user testing.^15^ However, the Medusa Advisory Board has discussed whether more contextual data should be provided, to allow clinicians to make better informed risk:benefit decisions. While a large amount of additional information could be provided, each additional piece of information increases the complexity of the compatibility section and thus the risk of misinterpretation. Given the lack of research evidence, the optimum combination of drug compatibility information to include in Medusa is unclear.

This project therefore aimed to develop a set of evidence-based proposals for the future drug compatibility information provided in Medusa. Specific objectives included:

1. Estimating the frequency with which Medusa users are involved with the co-infusion of intravenous drugs.
2. Identifying the drugs most commonly co-infused by Medusa users.
3. Identifying the types of information potentially relevant to intravenous drug compatibility that are most important for Medusa users.
4. Estimating the frequency with which Medusa users access its compatibility information.
5. Measuring the usability of the current Medusa compatibility information.
6. Developing subject expert recommendations for the drug compatibility information that should be included in Medusa, including the drugs for which this information should be provided, the type of evidence that should underpin it and the types of information that should be included.

## Methods

A convergent parallel design was used, with an anonymous, cross-sectional online survey used to collect the views of Medusa users on their use of the current compatibility section and the importance of the different types of information that it might contain (objectives 1–5). This survey is reported in accordance with the Consensus-Based Checklist for Reporting of Survey Studies (online supplemental table S1).^16^ The views of a varied panel of health professionals with differing expertise relevant to the administration of intravenous medicines were investigated using a three-round Delphi technique (objective 6), which is widely used to resolve clinical uncertainty in the absence of definitive evidence.^17^ It is designed to develop consensus between experts in relation to their agreement with statements describing the topic under investigation. It is iterative, with the experts asked to rate their agreement with each statement through several sequential rounds.^17^,^19^ The Delphi project is reported in accordance with the Guidance on Conducting and REporting DElphi Studies checklist (online supplemental table S2).^17^ In both studies, participants were informed that it related to the administration of two or more intravenous drugs at the same time via the same intravenous line or lumen, so they mix before entering the bloodstream.

This project was categorised as a service evaluation of Medusa by Imperial College Healthcare NHS Trust; therefore, review by a research ethics committee was not required. However, the project applied the same ethical standards as would be expected by an ethics committee, considering aspects such as participant well-being, informed consent and confidentiality. The design and project materials were therefore reviewed and approved by the Pharmacy Department Quality and Safety Committee and the Clinical Audit Team of Imperial College Healthcare NHS Trust on 4 May 2023 (number 863).

### User survey

#### User survey: development

The survey was designed using Jisc Online Surveys (www.onlinesurveys.ac.uk) (online supplemental appendix S2). It began with participant information and a question confirming consent to participate. Participants were then asked how often, and for which drugs, they were involved with co-infusion over the past year (objectives 1 and 2). The potential drugs were classified into 17 drugs/groups based on previous research.^45 7^,^9 20^ Next, the survey asked participants to rate the importance of 13 types of information potentially relevant to intravenous drug compatibility using a five-point rating scale (objective 3). The 13 types of information were derived from the literature^11^,^13^ and discussion with members of the Medusa Advisory Board and editorial team. Next, the survey asked how often participants used Medusa and its compatibility information section, before asking their agreement with five statements regarding the usability of the Medusa compatibility section using a five-point scale (objectives 4 and 5). These five statements were based on the five most relevant domains of Rosenbaum’s user experience framework.^21^ A free-text question asked how the Medusa compatibility section could be improved. The final page collected data on participants’ professional experience. The survey was pre-tested with two pharmacist members of the Medusa editorial team using the talk-aloud technique.

#### User survey: participants and recruitment

All Medusa users were eligible to complete the survey. A link to the survey was placed on the first page seen after logging into the Medusa website from July to September 2023. Potential participants were also invited via an email including brief project information and a link to the survey, which was distributed via members of the Medusa Advisory Board and other professional networks. The survey was closed in September 2023 as the rate of recruitment at this time was very slow and all potential routes for advertising the study had been used. Based on a conservative estimate of the coefficient of variation typically seen for five-point Likert scale survey items (0.5), a target sample size of 385 participants was chosen to enable estimation of the population’s rating of the importance of the 13 types of information potentially relevant to intravenous drug compatibility with a 5% margin of error.^22^

#### User survey: analysis

Statistical analysis was performed using SPSS (IBM, V.27). Participant characteristics were summarised using descriptive statistics. CIs of proportions were calculated using the Wilson score method.^23^ Rating scale responses were converted to integers (1–5) and summarised using the median and IQR. Ratings of different subgroups of participants were compared using Mann-Whitney U tests. Ratings of different questions were compared using Friedman tests followed by Dunn-Bonferroni post hoc tests. Recurrent themes in free-text comments were summarised.

### Delphi project

#### Delphi project: development

36 statements describing all the compatibility information that might be included in Medusa were developed from the literature^11^,^13^ and discussion with members of the Medusa Advisory Board and editorial team (table 1). An online survey was designed using Jisc Online Surveys (www.onlinesurveys.ac.uk) to assess participants’ agreement with each of the 36 statements in round 1 (online supplemental appendix 3). It began with participant information and a question confirming consent to participate. The survey then asked participants to rate their agreement with each statement using a six-point rating scale. This ensured participants expressed a positive or negative opinion by avoiding a neutral mid-point.^18^ Free-text questions asked participants to explain their reported levels of agreement and to suggest additional compatibility information that should be included. The survey was pre-tested with one potentially eligible participant to ensure that questions were correctly understood and easily completed. The talk-aloud technique was used in an online meeting. Subsequently, various revisions were made to wording throughout the survey.

**Table 1:** Initial Delphi statements describing all the possible information that might be included in the Medusa compatibility section

Subject area	Statement
Drugs for which compatibility information should be provided*	Only IV guides on Medusa for an AGREED LIST of relevant primary drugs should include compatibility information
	All IV drug guides on Medusa for individual primary drugs given by CONTINUOUS INFUSION should include information on compatibility
	All IV drug guides on Medusa for individual primary drugs given by SHORT INFUSION should include information on compatibility
	All IV drug guides on Medusa for individual primary drugs given by INJECTION should include information on compatibility
	Only secondary drugs from an AGREED LIST of relevant drugs should be included
	All secondary drugs given by CONTINUOUS INFUSION should be included
	All secondary drugs given by SHORT INFUSION should be included
	All secondary drugs given by INJECTION should be included
Information on compatibility or incompatibility	The Medusa compatibility section should include information on drug combinations that are known to be COMPATIBLE
	The Medusa compatibility section should include information on drug combinations that are known to be INCOMPATIBLE
	The Medusa compatibility section should list relevant drug combinations where compatibility is NOT KNOWN
Type of underpinning evidence: analytical method	The Medusa compatibility section should state that drug combinations are COMPATIBLE based on reports of visual inspection of the mixture for signs of incompatibility
	The Medusa compatibility section should state that drug combinations are INCOMPATIBLE based on reports of visual inspection of the mixture for signs of incompatibility
	The Medusa compatibility section should state that drug combinations are COMPATIBLE based on data from scientific instruments that detect particles
	The Medusa compatibility section should state that drug combinations are INCOMPATIBLE based on data from scientific instruments that detect particles
	The Medusa compatibility section should state that drug combinations are COMPATIBLE based on chemical analysis
	The Medusa compatibility section should state that drug combinations are INCOMPATIBLE based on chemical analysis
Drug concentration information	The Medusa compatibility section should only state that a pair of drugs are compatible when the supporting data relate to drug concentrations used in practice
	The Medusa compatibility section should state the concentrations at which two drugs are compatible
Infusion solution information	The Medusa compatibility section should only state that a pair of drugs are compatible when the supporting data relate to infusion solutions used in practice
	The Medusa compatibility section should state the infusion solutions in which two drugs are compatible
Timeframe information	The Medusa compatibility section should only state that a pair of drugs are compatible when this is over a time scale relevant to practice
	The Medusa compatibility section should state for how long a pair of drugs are COMPATIBLE
	The Medusa compatibility section should state how long it takes a pair of drugs to become INCOMPATIBLE
Environmental conditions information	The Medusa compatibility section should only state that a pair of drugs are compatible when the supporting data relate to temperatures relevant to administration (not storage) in practice
	The Medusa compatibility section should state the temperatures at which two drugs are compatible
	The Medusa compatibility section should only state that a pair of drugs are compatible when the supporting data relate to administration in light (not protected from light)
	The Medusa compatibility section should state whether two drugs are compatible in light or darkness
IV container/line information	The Medusa compatibility section should only state that a pair of drugs are compatible when the supporting data relate to an IV container and/or IV line material used in practice
	Where relevant, the Medusa compatibility section should state what the IV container and/or IV line containing a mixture of two drugs should be made from (eg, glass, PVC)
Type of underpinning evidence: point of contact	The Medusa compatibility section should state that a pair of drugs are compatible when the supporting data relate to mixing in the same container (eg, bag, syringe)
	The Medusa compatibility section should state that a pair of drugs are compatible when the supporting data relate to mixing in the IV line
	The Medusa compatibility section should describe whether compatibility is for mixing in the IV line or in the same container (eg, bag, syringe)
Additional information provision	The Medusa compatibility section should include the pH of the individual drugs. (NB: Medusa also provides this pH in a separate section).
	The Medusa compatibility section should include details on where information came from, for example, references
	The Medusa compatibility section should include information on how compatibility was measured, for example, visual inspection for cloudiness, chemical analysis etc.

These statements assume that information is only provided to support the co-infusion of drugs that meet for the first time in the intravenous administration set, where they mix before entering the bloodstream.

* A ‘primary drug’ was defined as the drug which is the main subject of a Medusa monograph, whereas a ‘secondary drug’ was defined as another drug included in the compatibility section of a primary drug’s Medusa monograph. For example, Medusa’s fentanyl monograph includes information on compatibility with epinephrine. In this situation, fentanyl is the ‘primary drug’ and epinephrine is the ‘secondary drug’. However, when considering the Medusa monograph for epinephrine (which includes compatibility information with fentanyl), epinephrine is the ‘primary drug’ and fentanyl is the ‘secondary drug’.

IV, intravenous; PVC, polyvinyl chloride.

**Figure 1:**
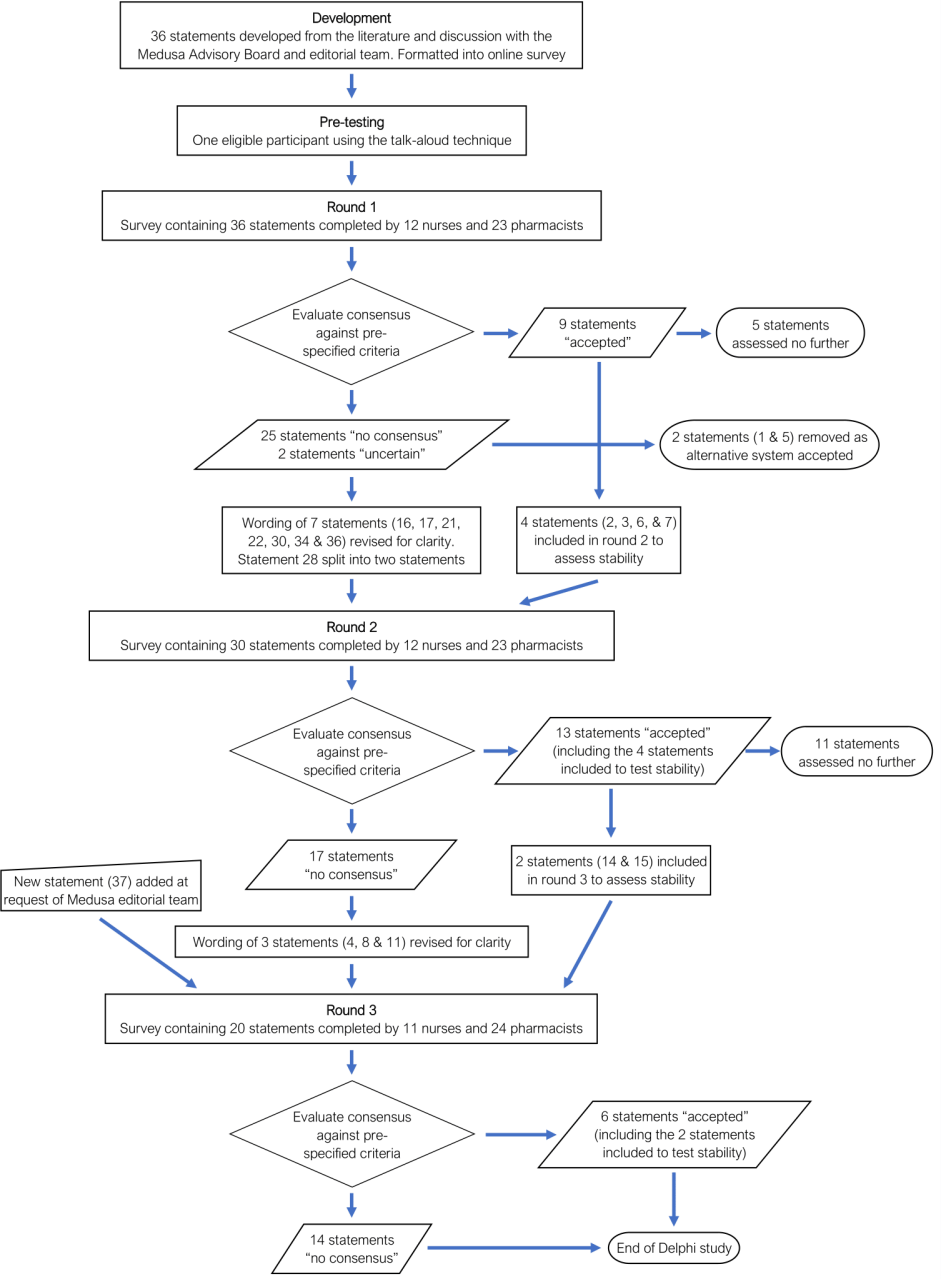
Flow chart illustrating the development and outcomes of the Delphi project.

The round 2 and 3 surveys used the same format, but excluded most statements that had been accepted or rejected in the previous round (figure 1 and online supplemental appendices 4 and 5). However, to assess response stability, selected statements were included despite being previously accepted. One additional statement was included in round 3 in response to a specific request from the Medusa editorial team (statement 37). Where necessary, the wording of selected round 2 and 3 statements was revised to improve clarity, in response to participants’ free-text comments (online supplemental tables S6 and S7). The round 2 and 3 surveys also included the agreement ratings for each statement and representative free-text quotations from the previous round’s responses, to summarise the level of agreement with each statement and the reasons for this. This enabled participants to reconsider their views in the light of other opinions, which can facilitate the development of consensus.^18 19^

#### Delphi project: participants and recruitment

Initial responses to the user survey indicated that nurses and pharmacists were the two professions that commonly use Medusa. Therefore, participants were eligible for inclusion in the Delphi project if they were a registered nurse or pharmacist currently practising in the NHS with professional expertise in an area of relevance to the compatibility of injectable medicines. Eligible participants were identified via the Medusa Advisory Board and other professional networks, as well as by snowballing. Purposive sampling was used to obtain a diverse sample in terms of professional background, relevant expertise and Advisory Board membership, with the aim of recruiting at least 8–15 participants from each profession in each round.^18^ Potential participants were invited to participate via an email which included brief project information and a link to the online survey. Up to three email reminders were sent for each round of the survey.

#### Delphi project: analysis

Statistical analysis was performed using SPSS (IBM, V.27). Agreement rating scale responses were converted to integers between 1 (strongly disagree) and 6 (strongly agree) and summarised using the median and IQR. The following definitions were pre-specified:

- A statement was ‘accepted’ if the medians of both nurses’ and pharmacists’ responses were ≥5 with IQRs ≤1.75.^18^
- A statement was ‘rejected’ if the medians of both nurses’ and pharmacists’ responses were ≤2 with IQRs ≤1.75.
- A statement was ‘uncertain’ if the median of nurses’ and/or pharmacists’ responses was in the range 2–5, with both professions’ IQR ≤1.75.
- ‘No consensus’ about a statement existed when the above criteria were not met.

To manage participant burden, it was pre-specified that no more than three rounds would be completed, even if there was uncertainty or no consensus about some statements. The responses of nurses and pharmacists were considered separately as each profession has a different role in the administration of intravenous medicines and so might have different perspectives. The stability of participants’ responses was assessed using the Wilcoxon signed rank test. Free-text responses were summarised and used to revise the wording of statements (where necessary) and feedback to participants in the following Delphi round.

### Patient and public involvement

As this evaluation focused on the information needs of healthcare professionals, there were no patient and public involvement activities.

## Results

### User survey

In total, 142 participants completed the survey, all of whom were either nurses, midwives or pharmacists (table 2). 112 participants (79%, 95% CI 71 to 85%) used Medusa at least once a week. 97 participants (68%, 95% CI 60 to 75%) were involved in the co-infusion of intravenous drugs at least once a week (objective 1), but only 60 (42%, 95% CI 34 to 51%) used the Medusa compatibility section this often (objective 4). There is no information on the characteristics of Medusa users, but the majority usage by nurses reported here is thought to be typical.

**Table 2:** Characteristics of user survey participants (objectives 1 and 4)

	Nurses or midwives	Pharmacists
Number of participants (%)*	114 (80.3%)	28 (19.7%)
Median years qualified (IQR)	11 (6–20)	10 (5 to 20)
Frequency (%) of involvement with the use of IV drugs via the same IV line or lumen over the past year†	Every day: 49 (43.0%, 95% CI 34.3 to 52.2%)Every week: 35 (30.7%, 95% CI 23.0 to 39.7%)Every month: 8 (7.0%, 95% CI 3.6 to 13.2%)Less than once a month: 17 (14.9%, 95% CI 9.5 to 22.6%)Never: 5 (4.4%, 95% CI 1.9 to 9.9%)	Every day: 8 (28.6%, 95% CI 15.3 to 47.1%)Every week: 5 (17.9%, 95% CI 7.9 to 35.6%)Every month: 8 (28.6%, 95% CI 15.3 to 47.1%)Less than once a month: 4 (14.3%, 95% CI 5.7 to 31.5%)Never: 3 (10.7%, 95% CI 3.7 to 27.2%)
Frequency (%) of Medusa use over the past year†	Every day: 46 (40.4%, 95% CI 31.8 to 49.5%)Every week: 51 (44.7%, 95% CI 35.9 to 53.9%)Every month: 12 (10.5%, 95% CI 6.1 to 17.5%)Less than once a month: 4 (3.5%, 95% CI 1.4 to 8.7%)Never: 1 (0.9%, 95% CI 0.2 to 4.8%)	Every day: 4 (14.3%, 95% CI 5.7 to 31.5%)Every week: 11 (39.3%, 95% CI 23.6 to 57.6%)Every month: 11 (39.3%, 95% CI 23.6 to 57.6%)Less than once a month: 2 (7.1%, 95% CI 2.0 to 22.6%)Never: 0 (0.0%, 95% CI 0.0 to 12.1%)
Frequency (%) of Medusa compatibility section use over the past year†	Every day: 16 (14.0%, 95% CI 8.8 to 21.6%)Every week: 36 (31.6%, 95% CI 23.8 to 40.6%)Every month: 18 (15.8%, 95% CI 10.2 to 23.6%)Less than once a month: 20 (17.5%, 95% CI 11.7 to 25.6%)Never: 5 (4.4%, 95% CI 1.9 to 9.9%)I didn’t know it existed: 19 (16.7%, 95% CI 10.9 to 24.6%)	Every day: 1 (3.6%, 95% CI 0.6 to 17.7%)Every week: 7 (25.0%, 95% CI 12.7 to 43.4%)Every month: 15 (53.6%, 95% CI 35.8 to 70.5%)Less than once a month: 4 (14.3%, 95% CI 5.7 to 31.5%)Never: 0 (0.0%, 95% CI 0.0 to 12.1%)I didn’t know it existed: 1 (3.6%, 95% CI 0.6 to 17.7%)
Frequency (%) of adult clinical specialities worked over the past year†‡	Ambulance service: 2 (1.8%, 95% CI 0.5 to 6.2%)Emergency department: 8 (7.0%, 95% CI 3.6 to 13.2%)Inpatient wards: 42 (36.8%, 95% CI 28.6 to 46.0%)Critical or intensive care: 44 (38.6%, 95% CI 30.2 to 47.8%)Theatre or recovery: 6 (5.3%, 95% CI 2.4 to 11.0%)Ambulatory or day care: 3 (2.6%, 95% CI 0.9 to 7.5%)Outpatients: 8 (7.0%, 95% CI 3.6 to 13.2%)Hospital at home: 0 (0.0%, 95% CI 0.0 to 3.3%)Community services: 2 (1.8%, 95% CI 0.5 to 6.2%)Mental health: 1 (0.9%, 95% CI 0.2 to 4.8%)Other: 5 (4.4%, 95% CI 1.9 to 9.9%)	Ambulance service: 0 (0.0%, 95% CI 0.0 to 12.1%)Emergency department: 1 (3.6%, 95% CI 0.6 to 17.7%)Inpatient wards: 18 (64.3%, 95% CI 45.8 to 79.3%)Critical or intensive care: 8 (28.6%, 95% CI 15.3 to 47.1%)Theatre or recovery: 6 (21.4%, 95% CI 10.2 to 39.5%)Ambulatory or day care: 3 (10.7%, 95% CI 3.7 to 27.2%)Outpatients: 5 (17.9%, 95% CI 7.9 to 35.6%)Hospital at home: 0 (0.0%, 95% CI 0.0 to 12.1%)Community services: 0 (0.0%, 95% CI 0.0 to 12.1%)Mental health: 1 (3.6%, 95% CI 0.6 to 17.7%)Other: 3 (10.7%, 95% CI 3.7 to 27.2%)
Frequency (%) of paediatric clinical specialities worked over the past year†‡	Ambulance service: 2 (1.8%, 95% CI 0.5 to 6.2%)Emergency department: 6 (5.3%, 95% CI 2.4 to 11.0%)Inpatient wards: 19 (16.7%, 95% CI 10.9 to 24.6%)Critical or intensive care: 17 (14.9%, 95% CI 9.5 to 22.6%)Theatre or recovery: 5 (4.4%, 95% CI 1.9 to 9.9%)Ambulatory or day care: 1 (0.9%, 95% CI 0.2 to 4.8%)Outpatients: 3 (2.6%, 95% CI 0.9 to 7.5%)Hospital at home: 1 (0.9%, 95% CI 0.2 to 4.8%)Community services: 1 (0.9%, 95% CI 0.2 to 4.8%)Mental health: 0 (0.0%, 95% CI 0.0 to 3.3%)Other: 0 (0.0%, 95% CI 0.0 to 3.3%)	Ambulance service: 0 (0.0%, 95% CI 0.0 to 12.1%)Emergency department: 0 (0.0%, 95% CI 0.0 to 12.1%)Inpatient wards: 7 (25.0%, 95% CI 12.7 to 43.4%)Critical or intensive care: 5 (17.9%, 95% CI 7.9 to 35.6%)Theatre or recovery: 1 (3.6%, 95% CI 0.6 to 17.7%)Ambulatory or day care: 0 (0.0%, 95% CI 0.0 to 12.1%)Outpatients: 3 (10.7%, 95% CI 3.7 to 27.2%)Hospital at home: 0 (0.0%, 95% CI 0.0 to 12.1%)Community services: 0 (0.0%, 95% CI 0.0 to 12.1%)Mental health: 0 (0.0%, 95% CI 0.0 to 12.1%)Other: 1 (3.6%, 95% CI 0.6 to 17.7%)

* Percentages are based on all participants.

† Percentages are based on profession.

‡ Participants could select more than one specialty.

A wide range of types of drug were reported to be co-infused (table 3), with intravenous fluids, antibiotics and analgesics all reported by at least half of participants (objective 2).

**Table 3:** Drugs and drug classes Medusa users reported administering or seeing administered via the same intravenous line or lumen as another drug over the previous year (objective 2)

Drug class	Frequency (%)
Intravenous fluids	116 (81.7%, 95% CI 74.5 to 87.2%)
Antibiotics	85 (59.9%, 95% CI 51.6 to 67.6%)
Analgesics	81 (57.0%, 95% CI 48.8 to 64.9%)
Insulin	70 (49.3%, 95% CI 41.2 to 57.4%)
Electrolytes	69 (48.6%, 95% CI 40.5 to 56.7%)
Sedatives	61 (43.0%, 95% CI 35.1 to 51.2%)
Vasopressors and inotropes	56 (39.4%, 95% CI 31.8 to 47.7%)
Diuretics	48 (33.8%, 95% CI 26.5 to 41.9%)
Muscle relaxants	42 (29.6%, 95% CI 22.7 to 37.5%)
Vasopressin (argipressin)	42 (29.6%, 95% CI 22.7 to 37.5%)
Steroids	39 (27.5%, 95% CI 20.8 to 35.3%)
Anti-emetics	36 (25.4%, 95% CI 18.9 to 33.1%)
Heparin	36 (25.4%, 95% CI 18.9 to 33.1%)
Cardioactive drugs	35 (24.6%, 95% CI 18.3 to 32.3%)
Acid suppressants	31 (21.8%, 95% CI 15.8 to 29.3%)
Antifungals	30 (21.1%, 95% CI 15.2 to 28.6%)
Antivirals	28 (19.7%, 95% CI 14.0 to 27.0%)
Tranexamic acid	26 (18.3%, 95% CI 12.8 to 25.5%)
Anti-epilepsy drugs	25 (17.6%, 95% CI 12.2 to 24.7%)
Acetylcysteine	20 (14.1%, 95% CI 9.3 to 20.8%)
Immunosuppressants	17 (12.0%, 95% CI 7.6 to 18.3%)
Aminophylline	17 (12.0%, 95% CI 7.6 to 18.3%)
Other*	4 (3.0%, 95% CI 1.1 to 7.0%)

Nine participants did not select any of these drug classes.

* Others included parenteral nutrition, immunoglobulin and octreotide.

Participants considered all 13 types of information to be important when deciding how to manage a patient who may need co-infusion of intravenous drugs, as the median score for all categories was either important or very important (table 4). Nurses and midwives rated information on how long drugs are compatible, temperature, light/darkness and how compatibility is measured as more important than pharmacists. Using the data from all participants, there were several significant differences between the importance rating of the 13 types of information, suggesting that participants considered information on whether drugs are compatible or incompatible, and the concentrations and diluents in which this occurs, to be most important (online supplemental table S3, objective 3).

**Table 4:** Medusa users’ rating of the importance of different types of information when deciding how to manage a patient who may need intravenous drugs administered via the same intravenous line or lumen (objective 3)

	Median (IQR) importance rating*			P value†
	All participants (n=136)‡	Nurses or midwives (n=108)‡	Pharmacists (n=28)	
Which drugs are compatible with each other	5 (5–5)	5 (5–5)	5 (4–5)	0.141
Which drugs are incompatible with each other	5 (5–5)	5 (5–5)	5 (5–5)	0.717
Drug concentrations where drugs are compatible	5 (4–5)	5 (4–5)	4 (4–5)	0.097
Infusion fluids in which drugs are compatible	5 (5–5)	5 (5–5)	5 (4–5)	0.285
For how long drugs are compatible	5 (4–5)	5 (4–5)	4 (4–4)	0.001
How long it takes drugs to become incompatible	4 (4–5)	5 (4–5)	4 (3–4)	0.011
Temperatures at which drugs are compatible	4 (3–5)	4 (4–5)	4 (2–4)	<0.001
Whether the drugs are compatible in light or darkness	4 (4–5)	5 (4–5)	4 (3–4)	<0.001
What the IV container (bag, syringe) and IV line should be made from (eg, glass, PVC)	4 (3–5)	4 (3–5)	4 (4–5)	0.309
Whether the information is for mixing in the IV line or in the same container (eg, bag, syringe)	5 (4–5)	5 (4–5)	4 (4–5)	0.118
How compatibility was measured eg, visual inspection for cloudiness, chemical analysis etc.	4 (3–5)	4 (3–5)	4 (2–4)	0.006
The pH of the drugs	4 (3–5)	4 (3–5)	4 (4–4)	0.309
Where the information came from eg, references	4 (3–5)	4 (3–5)	4 (4–4)	0.634

* Data presented are the median (IQR) of responses on a five-point Likert scale (1=very unimportant, 2=unimportant, 3=neither important nor unimportant, 4=important, 5=important).

† p values are for the comparison of nurses’ or midwives’ responses with pharmacists’ responses using the Mann-Whitney U test.

‡ Six nurses did not answer these questions.

IV, intravenous.

Overall, participants considered the compatibility section to be usable in all five domains, as the median score for all categories was either agree or strongly agree (table 5, objective 5). There were no significant differences between nurses and midwives, and pharmacists. However, using the data from all participants, there were significant differences between agreement with the five statements (Friedman test χ^2^(4), = 64.374, p<0.001), with the statements ‘it is useful’ and ‘it is intended for someone doing a job like me’ having significantly greater agreement than the other three statements (p<0.031 in each case).

**Table 5:** Medusa users’ agreement with various statements relating to their user experience of the Medusa compatibility section (objective 5)

	Median (IQR) agreement*			P value†
	All participants	Nurses or midwives	Pharmacists	
It is easy to find‡	4 (3–4)	4 (3–4)	4 (3–5)	0.245
It is easy to understand‡	4 (3–4)	4 (3–4)	4 (3–5)	0.756
It is easy to use‡	4 (3–4)	4 (3–4)	4 (3–5)	0.821
It is useful‡	4 (4–5)	4 (4–5)	4 (4–5)	0.476
It is intended for someone doing a job like me	4 (4–5)	4 (4–5)	4 (4–5)	0.801

* Data presented are the median (IQR) of responses on a five-point Likert scale (1=strongly disagree, 2=disagree, 3=neither agree nor disagree, 4=agree, 5=strongly agree). Participants could also respond “I haven’t seen it”—these data were excluded from the median calculation.

† p values are for the comparison of nurses’ or midwives’ responses with pharmacists’ responses using the Mann-Whitney U test.

‡ No response from one participant.

The following suggestions to improve the Medusa compatibility section were commonly seen in free-text responses: provide the information as a chart or searchable database, use of features such as colour coding to make it easier to understand the information, provide compatibility information on more drug combinations, raise awareness of the existence of the section and when it should be used, and provide compatibility information earlier in the monograph.

### Delphi project

In total, 59 health professionals were invited to participate, and 41 (69%) took part in at least one Delphi round (online supplemental table S4). 26 participants completed all three rounds; however, analysis was based on all responses submitted to each round. Each round received responses from 35 participants. The characteristics of participants in each round are summarised in table 6. Approximately one-third of participants in each round were nurses, with the remaining participants being pharmacists. Over half of participants in each round were not Medusa Advisory Board members and they had a wide range of relevant expertise developed over many years of experience.

**Table 6:** Characteristics of the participants who took part in each round of the Delphi process

		Round 1	Round 2	Round 3
Nurses	Number of participants	12	12	11
	Median years’ experience (IQR)	18 (10–30)	22 (10–32)	18 (9–26)
	Number of advisory board members	3	3	3
	Specialities	Education (n=1)Infection prevention (n=1)Intensive care (n=4)Intensive care (education) (n=4)Intravenous access (n=1)OPAT (n=1)	Community IV therapy (n=1)Education (n=1)Infection prevention (n=1)Injectable medication safety (n=2)Intensive care (n=3)Intensive care (education) (n=4)	Community IV therapy (n=1)Education (n=1)Injectable medication safety (n=2)Intensive care (n=4)Intensive care (education) (n=2)Intravenous access (n=1)
Pharmacists	Number of participants	23	23	24
	Median years’ experience (IQR)	20 (9–29)	23 (9–30)	22 (10–30)
	Number of advisory board members	9	11	11
	Specialities	Antimicrobials (n=2)Clinical pharmacy (n=3)ePMA (n=2)Intensive care (n=6)Medicines information (n=1)Medicines safety (n=3)Oncology (n=1)OPAT (n=1)Paediatrics (n=1)Palliative care (n=1)Quality assurance (n=2)	Antimicrobials (n=2)Clinical pharmacy (n=2)ePMA (n=2)Intensive care (n=7)Medicines information (n=1)Medicines safety (n=3)OPAT (n=1)Paediatrics (n=2)Palliative care (n=1)Quality assurance (n=2)	Antimicrobials (n=2)Clinical pharmacy (n=3)ePMA (n=2)Intensive care (n=7)Medicines information (n=1)Medicines safety (n=3)OPAT (n=1)Paediatrics (n=2)Palliative care (n=1)Quality assurance (n=2)

ePMA, electronic prescribing and medicines administration; IV, intravenous; OPAT, outpatient parenteral antibiotic therapy.

Figure 1 illustrates the development of the project over the three rounds and numerical results for each round are shown in online supplemental tables S5–S7. After the three Delphi rounds had been completed, 22 statements had been accepted and there was no consensus about the remaining 16 statements (table 7, objective 6).

**Table 7:** Summary of the outcome of each round of the Delphi process for each consensus statement. Statements that were ultimately accepted are highlighted in bold text (objective 6)

		Round 1 outcome	Round 2 outcome	Round 3 outcome
1	Only IV guides on Medusa for an AGREED LIST of relevant primary drugs should include compatibility information	No consensus	–	–
**2**	**All IV drug guides on Medusa for individual primary drugs given by CONTINUOUS INFUSION should include information on compatibility**	**Accepted**	**Accepted**	**–**
**3**	**All IV drug guides on Medusa for individual primary drugs given by SHORT INFUSION should include information on compatibility**	**Accepted**	**Accepted**	**–**
4	R1&2: All IV drug guides on Medusa for individual primary drugs given by INJECTION should include information on compatibilityR3: All IV guides on Medusa for drugs given by INJECTION ONLY should include information on their compatibility with other drugs	No consensus	No consensus	No consensus
5	Only secondary drugs from an AGREED LIST of relevant drugs should be included	No consensus	–	–
**6**	**All secondary drugs given by CONTINUOUS INFUSION should be included**	**Accepted**	**Accepted**	**–**
**7**	**All secondary drugs given by SHORT INFUSION should be included**	**Accepted**	**Accepted**	**–**
8	R1&2: All secondary drugs given by INJECTION should be includedR3: All IV guides on Medusa for drugs given by CONTINUOUS OR SHORT INFUSION should include information on their compatibility with drugs given by INJECTION ONLY	No consensus	No consensus	No consensus
**9**	**The Medusa compatibility section should include information on drug combinations that are known to be COMPATIBLE**	**Accepted**	**–**	**–**
**10**	**The Medusa compatibility section should include information on drug combinations that are known to be INCOMPATIBLE**	**Accepted**	**–**	**–**
11	R1&2: The Medusa compatibility section should list relevant drug combinations where compatibility is NOT KNOWNR3: The Medusa compatibility section should list COMMON, IMPORTANT drug combinations where compatibility is NOT KNOWN	No consensus	No consensus	No consensus
12	The Medusa compatibility section should state that drug combinations are COMPATIBLE based on reports of visual inspection of the mixture for signs of incompatibility	No consensus	No consensus	No consensus
**13**	**The Medusa compatibility section should state that drug combinations are INCOMPATIBLE based on reports of visual inspection of the mixture for signs of incompatibility**	**Accepted**	**–**	**–**
**14**	**The Medusa compatibility section should state that drug combinations are COMPATIBLE based on data from scientific instruments that detect particles**	**No consensus**	**Accepted**	**Accepted**
**15**	**The Medusa compatibility section should state that drug combinations are INCOMPATIBLE based on data from scientific instruments that detect particles**	**No consensus**	**Accepted**	**Accepted**
**16**	**R1: The Medusa compatibility section should state that drug combinations are COMPATIBLE based on chemical analysisR2: The Medusa compatibility section should state that drug combinations are COMPATIBLE based on chemical analysis that can detect degradation of drugs that does NOT result in cloudiness or particle formation**	**No consensus**	**Accepted**	**–**
**17**	**R1: The Medusa compatibility section should state that drug combinations are INCOMPATIBLE based on chemical analysisR2: The Medusa compatibility section should state that drug combinations are INCOMPATIBLE based on chemical analysis that can detect degradation of drugs that does NOT result in cloudiness or particle formation**	**No consensus**	**Accepted**	**–**
**18**	**The Medusa compatibility section should only state that a pair of drugs are compatible when the supporting data relate to drug concentrations used in practice**	**Uncertain**	**No consensus**	**Accepted**
**19**	**The Medusa compatibility section should state the concentrations at which two drugs are compatible**	**No consensus**	**No consensus**	**Accepted**
**20**	**R1: The Medusa compatibility section should only state that a pair of drugs are compatible when the supporting data relate to infusion solutions used in practiceR2&3: The Medusa compatibility section should only state that a pair of drugs are compatible when the supporting data relate to infusion solutions (diluents) used in practice**	**No consensus**	**No consensus**	**Accepted**
**21**	**R1: The Medusa compatibility section should state the infusion solutions in which two drugs are compatibleR2: The Medusa compatibility section should state the infusion solutions (diluents) in which two drugs are compatible**	**No consensus**	**Accepted**	** –**
**22**	**The Medusa compatibility section should only state that a pair of drugs are compatible when this is over a time scale relevant to practice**	**No consensus**	**Accepted**	**–**
**23**	**The Medusa compatibility section should state for how long a pair of drugs are COMPATIBLE**	**No consensus**	**No consensus**	**Accepted**
24	The Medusa compatibility section should state how long it takes a pair of drugs to become INCOMPATIBLE	No consensus	No consensus	No consensus
**25**	**The Medusa compatibility section should only state that a pair of drugs are compatible when the supporting data relate to temperatures relevant to administration (not storage) in practice**	**No consensus**	**Accepted**	**–**
26	The Medusa compatibility section should state the temperatures at which two drugs are compatible	No consensus	No consensus	No consensus
27	The Medusa compatibility section should only state that a pair of drugs are compatible when the supporting data relate to administration in light (not protected from light)	No consensus	No consensus	No consensus
28	The Medusa compatibility section should state whether two drugs are compatible in light or darkness	Uncertain	–	–
28.1	The Medusa compatibility section should state when two drugs are compatible in light	–	No consensus	No consensus
28.2	The Medusa compatibility section should state when two drugs are only compatible in darkness	–	No consensus	No consensus
29	The Medusa compatibility section should only state that a pair of drugs are compatible when the supporting data relate to an IV container and/or IV line material used in practice	No consensus	No consensus	No consensus
**30**	**R1: Where relevant, the Medusa compatibility section should state what the IV container and/or IV line containing a mixture of two drugs should be made from (eg, glass, PVC)R2: If there are specific requirements, the Medusa compatibility section should state what the IV container and/or IV line containing a mixture of two drugs should be made from (eg, glass, PVC)**	**No consensus**	**Accepted**	**–**
**31**	**The Medusa compatibility section should state that a pair of drugs are compatible when the supporting data relate to mixing in the same container (eg, bag, syringe)**	**No consensus**	**Accepted**	**–**
**32**	**The Medusa compatibility section should state that a pair of drugs are compatible when the supporting data relate to mixing in the IV line**	**Accepted**	**–**	**–**
**33**	**The Medusa compatibility section should describe whether compatibility is for mixing in the IV line or in the same container (eg, bag, syringe)**	**Accepted**	**–**	**–**
34	R1: The Medusa compatibility section should include the pH of the individual drugs. (NB: Medusa also provides this pH in a separate section).R2&3: The Medusa compatibility section should include the pH of the individual drugs in ADDITION to it already being provided in the separate pH section.	No consensus	No consensus	No consensus
35	The Medusa compatibility section should include details on where information came from, eg, references	No consensus	No consensus	No consensus
36	R1: The Medusa compatibility section should include information on how compatibility was measured, for example, visual inspection for cloudiness, chemical analysis etc.R2&3: The Medusa compatibility section should include information on how the compatibility data on which it is based was obtained, for example, visual inspection for cloudiness, chemical analysis etc.	No consensus	No consensus	No consensus
37*	In ADDITION to ‘Y-site’ compatibility information, the Medusa compatibility section should include information on the mixing of drugs in the SAME CONTAINER (eg, infusion bag, syringe)	–	–	No consensus

* New statement added for round 3 in response to specific request from Medusa editorial team.

IV, intravenous; R1, round 1; R2, round 2; R3, round 3.

Participants who completed both rounds 1 and 2 (n=28) agreed significantly more strongly with statement 2 in the second round than the first round (p=0.021), although this statement was accepted in both rounds. There was no significant difference in the level of agreement with statements 3, 6 and 7 (p=0.747, p=0.052 and p=0.971, respectively). Among participants who completed both rounds 2 and 3 (n=32), there was no significant difference in the level of agreement with statements 14 and 15 (p=0.634 and p=0.897, respectively).

## Discussion

Medusa users report that a wide range of drugs continue to be co-infused on a regular basis (tables2  3, objectives 1 and 2), confirming the clinical importance of easily available, relevant and understandable information on intravenous drug compatibility. It is therefore reassuring that the majority of users reported that the current Medusa compatibility section was easy to find, understand and use, while also providing useful information (table 5, objective 5). However, Medusa users also reported using its compatibility section less frequently than they co-infused intravenous drugs (objective 4). These users identified information on drugs that are compatible or incompatible, and the concentrations and diluents in which this occurs, to be most important (online supplemental table S3, objective 3). The expert panel also agreed that these topics should be included in Medusa (table 7, objective 6), suggesting that they represent the most relevant information on intravenous drug compatibility for clinicians. The expert panel also identified information on time frames, specific requirements for the intravenous line and whether supporting data relate to mixing in an intravenous line or container as important (table 7), but these were not identified by users (online supplemental table S3).

The expert panel agreed that such information should be provided for all drug pairs where both products are given by either continuous or short infusion; information for pairs where one of the drugs was given only by injection was unnecessary (table 7). The panel also agreed that a ‘compatible’ recommendation should be based on either chemical analysis or particle count data, whereas an ‘incompatible’ recommendation could also be based on visual inspection data. These data should relate to drug concentrations, diluents, time scales and temperatures used in practice, and data relating to drug mixing in the same container and/or the same intravenous line could be used to support compatible/incompatible recommendations for co-infusion.

Previous research has also identified that co-infusion of intravenous medicines is common in ICUs.^4 5^ In addition, the drug classes identified by the present evaluation as being commonly co-infused are similar to those reported in previous research, including antibiotics, analgesics, insulin, electrolytes, sedatives, vasopressors and inotropes.^47^,^9 20^ The expert panel recommendation to provide information for all pairs of drugs given by short or continuous infusion is in contrast to the recommendation in NPSA Alert 20,^10^ which stated that healthcare staff needed compatibility information for commonly used mixtures in specialist areas only. However, since this alert was published in 2007, clinicians have become more accustomed to having detailed electronic information available at the point of care and it is noteworthy that Oduyale *et al* found that the absence of compatibility information was the main challenge faced by ICU nurses in the Thames Valley region of England.^13^ In addition, Di Giorgi *et al* found that many databases do not provide information on all potential intravenous drug combinations.^11^

### Strengths and limitations

This is the first evaluation specifically designed to investigate clinicians’ intravenous drug compatibility information needs. Its strengths include the consideration of the perspectives of both Medusa users and subject experts, and the comparison of the opinions of both nurses and pharmacists. In the Delphi project, the target sample size was achieved and responses to previously accepted statements demonstrate that group stability was achieved once consensus was reached.^18^ The results are likely to be generalisable to Medusa users in the UK, but are unlikely to generalise internationally, due to the use of different medication prescribing and administration systems. Limitations include the sample size of the user survey, which did not achieve the target of 385 participants. However, as the coefficients of variation of the information importance ratings (table 4) were only 0.21–0.31 (less than the anticipated 0.5), the 136 participants who answered these questions still enabled a margin of error of 3.5–5.2%.^22^ The identification of some participants via the Medusa Advisory Board may have biased responses towards current information provision and more extensive pre-testing would provide greater assurance over the correct interpretation of the questions. Due to participation bias, the findings are likely to be more representative of clinicians with an interest in intravenous medicines administration.

### Recommendations

Given the tension between the findings of this evaluation and NPSA Alert 20, the Medusa Advisory Board should agree whether Medusa is intended as a quick reference guide for common drug combinations, or a comprehensive source of compatibility information. This will require consideration of both user needs and available resources. This overarching decision will guide the appropriate response to the following recommendations. The findings of this evaluation suggest that many users would like the compatibility section to be a comprehensive information source, so if this is not its intended aim, its more limited scope should be clearly communicated.

It is clear from previous research^47^,^9 20^ and the user survey (table 3) that a wide variety of drug combinations are frequently co-infused. In practice, Medusa currently provides compatibility information for most of these drug groups (online supplemental appendix 1). Despite this, a commonly suggested improvement from users was to provide information on more drug combinations, which may reflect the desire for information on combinations where compatibility is currently unknown. However, the expert panel recommended that compatibility information should only be provided for drug pairs where both drugs are given by either short or continuous infusion. Providing compatibility information for a larger number of drugs makes it more difficult to both update and use monographs. In this context, it is noteworthy that previous research found that compatibility charts were the easiest way to present this information, and these are restricted to a limited number of drugs.^11 13^ Therefore, if it is agreed that Medusa is intended as a quick reference guide for compatibility information, it should align its policy and practice to only provide information for the drugs listed in table 3 that are given by short or continuous infusion, while also signposting users to more comprehensive information sources. However, if Medusa is intended as a comprehensive source of compatibility information, this should be provided for all drug combinations where both drugs can be given by either short or continuous infusion. This may require a revised design for the compatibility section to ensure the necessary information can be easily found and understood. User testing will support this process and provide assurance of the performance of the final design,^15 24^ which is important because difficulties finding and understanding information in Medusa can contribute to medication errors.^25 26^

Findings from both the users and the expert panel suggest that information should continue to be provided on drug combinations that are known to be either compatible or incompatible. However, both groups also suggested that additional information should be provided on the concentration ranges and diluents in which drugs are compatible. Current Medusa editorial processes consider these issues when preparing guidance, but do not provide specific information. Therefore, consideration should be given to adding both compatible drug concentrations and diluent information, supported by user-tested design changes.

Finally, the current Medusa writing guidelines^27^ do not describe how authors should consider the underlying compatibility evidence when deciding whether to list a drug combination as compatible or incompatible. The addition of guidance based on the expert panel recommendations is therefore recommended, to support consistent evidence interpretation.

## Conclusion

The co-infusion of intravenous drugs is common, especially in critical care areas, so information to support clinical decision-making while administering multiple intravenous medicines continues to be important. The most relevant information includes drugs that are compatible or incompatible, and the concentrations and diluents in which this occurs. However, the expert panel’s recommendation to provide such information for all pairs of drugs given by short or continuous infusion contrasts with the more limited recommendation of NPSA Alert 20. The current Medusa compatibility section meets the needs of at least two-thirds of users, but greater clarity about whether it is intended as a quick reference guide or a comprehensive source of compatibility information will enable appropriate decisions to be made to improve the usability of this section even further. Further work is needed to understand the most effective ways to present such information so that it can be quickly understood and applied by clinicians. The co-infusion of intravenous medicines cannot always be avoided, but risks to patient safety can be minimised by providing clinicians with carefully designed and up-to-date drug compatibility information.

## Supplementary material

10.1136/bmjopen-2024-094211 online supplemental file 1

## Data Availability

No data are available.
