# Supplementary material for: Evaluation of NHS Injectable Medicines Guide users’ information needs related to the co-infusion of intravenous medicines: user survey and Delphi consensus study
Source: BMJ Open. 2025 May 30;15(5):e094211. doi: 10.1136/bmjopen-2024-094211 (PMC12128442; doi:10.1136/bmjopen-2024-094211)
Supplement: online supplemental file 1 [file bmjopen-15-5-s001.pdf]

BMJ Open

Evaluation of NHS Injectable Medicines Guide users' information needs related to the co-infusion of intravenous medicines via a user survey and Delphi consensus study

Virginia Aguado Lorenzo[1,2] and Matthew D. Jones (ORCID 0000-0002-2617-4098)[1,3]*

[1]Medusa NHS Injectable Medicines Guide, Imperial College Healthcare NHS Trust, London, UK.

[2]Pharmacy Department, Guy's and St Thomas' NHS Foundation Trust, London, UK

[3]Department of Life Sciences, University of Bath, Bath, UK.

*Corresponding author:

Department of Life Sciences, University of Bath, Bath, BA2 7AY, UK.

Telephone: +44 1225 383829, E-mail: M.D.Jones@bath.ac.uk

Table S1: the Consensus-Based Checklist for Reporting of Survey Studies (CROSS)[a]

Section/topic	Item	Item description	Comment	Location in manuscript
**Title and abstract**				
Title and abstract	1a	State the word “survey” along with a commonly used term in title or abstract to introduce the study’s design.		Title
	1b	Provide an informative summary in the abstract, covering background, objectives, methods, findings/results, interpretation/discussion, and conclusions.		Abstract
**Introduction**				
Background	2	Provide a background about the rationale of study, what has been previously done, and why this survey is needed.		Introduction
Purpose/aim	3	Identify specific purposes, aims, goals, or objectives of the study.		Introduction (final paragraph)
**Methods**				
Study design	4	Specify the study design in the methods section with a commonly used term (e.g., cross-sectional or longitudinal).		Methods
Data collection methods	5a	Describe the questionnaire (e.g., number of sections, number of questions, number and names of instruments used).		User survey: development
	5b	Describe all questionnaire instruments that were used in the survey to measure particular concepts. Report target population, reported validity and reliability information, scoring/classification procedure, and reference links (if any).	No specific instruments used	N/A
	5c	Provide information on pretesting of the questionnaire, if performed (in the article or in an online supplement). Report the method of pretesting, number of times questionnaire was pre-tested, number and demographics of participants used for pretesting, and the level of similarity of demographics between pre-testing participants and sample population.		User survey: development
	5d	Questionnaire if possible, should be fully provided (in the article, or as appendices or as an online supplement).		Online supplement, appendix 2
Sample characteristics	6a	Describe the study population (i.e., background, locations, eligibility criteria for participant inclusion in survey, exclusion criteria).		User survey: participants and recruitment
	6b	Describe the sampling techniques used (e.g., single stage or multistage sampling, simple random sampling, stratified sampling, cluster sampling, convenience sampling). Specify the locations of sample participants whenever clustered sampling was applied.		User survey: participants and recruitment

	6c	Provide information on sample size, along with details of sample size calculation.		User survey: participants and recruitment
	6d	Describe how representative the sample is of the study population (or target population if possible), particularly for population-based surveys.		Results: user survey
Survey administration	7a	Provide information on modes of questionnaire administration, including the type and number of contacts, the location where the survey was conducted (e.g., outpatient room or by use of online tools, such as SurveyMonkey).		User survey: development<br>User survey: participants and recruitment
	7b	Provide information of survey's time frame, such as periods of recruitment, exposure, and follow-up days.		User survey: participants and recruitment
	7c	Provide information on the entry process:<br>–>For non-web-based surveys, provide approaches to minimize human error in data entry.<br>–>For web-based surveys, provide approaches to prevent "multiple participation" of participants.	There was no way to prevent "multiple participation". This was assumed to be low risk, because advertising was only visible to health professionals and there was no incentive offered to participants.	N/A
Study preparation	8	Describe any preparation process before conducting the survey (e.g., interviewers' training process, advertising the survey).		User survey: participants and recruitment
Ethical considerations	9a	Provide information on ethical approval for the survey if obtained, including informed consent, institutional review board [IRB] approval, Helsinki declaration, and good clinical practice [GCP] declaration (as appropriate).		Methods<br>User survey: development
	9b	Provide information about survey anonymity and confidentiality and describe what mechanisms were used to protect unauthorized access.	The survey was anonymous and did not collect enough personal data (profession and years qualified) to identify any individual. The Jisc Online Surveys platform is GDPR compliant and certified to ISO 27001 standard. Access to the platform was restricted only to researchers. Data was stored on secure University of Bath servers.	Methods
Statistical analysis	10a	Describe statistical methods and analytical approach. Report the statistical software that was used for data analysis.		User survey: analysis
	10b	Report any modification of variables used in the analysis, along with reference (if available).	No modifications.	N/A
	10c	Report details about how missing data was handled. Include rate of missing items, missing data mechanism (i.e., missing completely at random [MCAR], missing at random [MAR] or missing not at random [MNAR]) and methods used to deal with missing data (e.g., multiple imputation).	No missing data for many items, and at most data missing for three participants in any one item. Therefore, too little missing data to estimate mechanism. Missing data ignored during subsequent analysis.	Missing items reports in results table footnotes
	10d	State how non-response error was addressed.	Multiple routes of advertising, short survey taking less than 10 minutes to complete, clear description of the survey given in the introduction.	N/A
	10e	For longitudinal surveys, state how loss to follow-up was addressed.	Not a longitudinal survey.	N/A

	10f	Indicate whether any methods such as weighting of items or propensity scores have been used to adjust for non-representativeness of the sample.	No adjustment.	N/A
	10g	Describe any sensitivity analysis conducted.	No sensitivity analysis.	N/A
**Results**				
Respondent characteristics	11a	Report numbers of individuals at each stage of the study. Consider using a flow diagram, if possible.	One stage study.	Results: user survey
	11b	Provide reasons for non-participation at each stage, if possible.	One stage study.	N/A
	11c	Report response rate, present the definition of response rate or the formula used to calculate response rate.	As the survey was advertised on the Medusa webpage, the number of potential participants who saw the advert is unknown. Therefore, the response rate cannot be calculated.	N/A
	11d	Provide information to define how unique visitors are determined. Report number of unique visitors along with relevant proportions (e.g., view proportion, participation proportion, completion proportion).	As described under item 7c above, there was no way to prevent "multiple participation", but this was assumed to be low risk.<br>In total, there were 534 visits to the survey information page. Of these, 244 (46%) participants began to complete the survey, of which 142 (27%) completed it.	N/A
Descriptive results	12	Provide characteristics of study participants, as well as information on potential confounders and assessed outcomes.		Table 2
Main findings	13a	Give unadjusted estimates and, if applicable, confounder-adjusted estimates along with 95% confidence intervals and p-values.	Not applicable to descriptive analytical approach.	N/A
	13b	For multivariable analysis, provide information on the model building process, model fit statistics, and model assumptions (as appropriate).	Multivariable analysis not used.	N/A
	13c	Provide details about any sensitivity analysis performed. If there are considerable amount of missing data, report sensitivity analyses comparing the results of complete cases with that of the imputed dataset (if possible).	No sensitivity analysis.	N/A
**Discussion**				
Limitations	14	Discuss the limitations of the study, considering sources of potential biases and imprecisions, such as non-representativeness of sample, study design, important uncontrolled confounders.		Strengths and limitations
Interpretations	15	Give a cautious overall interpretation of results, based on potential biases and imprecisions and suggest areas for future research.		Discussion<br>Conclusion
Generalizability	16	Discuss the external validity of the results.		Strengths and limitations
**Other sections**				

Role of funding source	17	State whether any funding organization has had any roles in the survey's design, implementation, and analysis.	Funding
Conflict of interest	18	Declare any potential conflict of interest.	Declaration of Conflicting Interests
Acknowledgements	19	Provide names of organizations/persons that are acknowledged along with their contribution to the research.	Acknowledgements

[a]Sharma A, Minh Duc NT, Luu Lam Thang T, et al. A Consensus-Based Checklist for Reporting of Survey Studies (CROSS). *J Gen Intern Med* 2021 2021/04/23. DOI: 10.1007/s11606-021-06737-1.

Table S2: the Guidance on Conducting and REporting DElphi Studies (CREDES) checklist[a]

Section/topic	Item	Item description	Comment	Location in manuscript
**Rationale for the choice of the Delphi technique**				
Justification	1	The choice of the Delphi technique as a method of systematically collating expert consultation and building consensus needs to be well justified. When selecting the method to answer a particular research question, it is important to keep in mind its constructivist nature.		Methods
**Planning and design**				
Planning and process	2	The Delphi technique is a flexible method and can be adjusted to the respective research aims and purposes. Any modifications should be justified by a rationale and be applied systematically and rigorously.	No modifications (e.g. face to face meetings)	N/A
Definition of consensus	3	Unless not reasonable due to the explorative nature of the study, an a priori criterion for consensus should be defined. This includes a clear and transparent guide for action on (a) how to proceed with certain items or topics in the next survey round, (b) the required threshold to terminate the Delphi process and (c) procedures to be followed when consensus is (not) reached after one or more iterations.		Delphi study: development<br>Delphi study: analysis
**Study conduct**				
Informational input	4	All material provided to the expert panel at the outset of the project and throughout the Delphi process should be carefully reviewed and piloted in advance in order to examine the effect on experts' judgements and to prevent bias.		Delphi study: development
Prevention of bias	5	Researchers need to take measures to avoid directly or indirectly influencing the experts' judgements. If one or more members of the research team have a conflict of interest, entrusting an independent researcher with the main coordination of the Delphi study is advisable.	The study was led and carried out by an independent academic researcher on secondment (MDJ)	N/A
Interpretation and processing of results	6	Consensus does not necessarily imply the 'correct' answer or judgement; (non)consensus and stable disagreement provide informative insights and highlight differences in perspectives concerning the topic in question.	Delphi findings are cross-referenced with user feedback before forming recommendations.	Discussion
External validation	7	It is recommended to have the final draft of the resulting guidance on best practice in palliative care reviewed and approved by an external board or authority before publication and dissemination.	Members of the Medusa editorial team reviewed a draft report of this study. The final report was reviewed by the Medusa Advisory Board in March 2024	N/A
**Reporting**				
Purpose and rationale	8	The purpose of the study should be clearly defined and demonstrate the appropriateness of the use of the Delphi technique as a method to achieve the research aim. A rationale for the choice of the Delphi technique as the most suitable method needs to be provided.		Methods
Expert panel	9	Criteria for the selection of experts and transparent information on recruitment of the expert panel, socio-demographic details including information on expertise regarding the topic in question, (non)response and response rates over the ongoing iterations should be reported.		Delphi study: participants and recruitment<br>Table 6

Description of the methods	10	The methods employed need to be comprehensible; this includes information on preparatory steps (How was available evidence on the topic in question synthesised?), piloting of material and survey instruments, design of the survey instrument(s), the number and design of survey rounds, methods of data analysis, processing and synthesis of experts' responses to inform the subsequent survey round and methodological decisions taken by the research team throughout the process.	Methods (including all sub-sections)
Procedure	11	Flow chart to illustrate the stages of the Delphi process, including a preparatory phase, the actual 'Delphi rounds', interim steps of data processing and analysis, and concluding steps.	Figure 1
Definition and attainment of consensus	12	It needs to be comprehensible to the reader how consensus was achieved throughout the process, including strategies to deal with non-consensus.	Delphi study: development
Results	13	Reporting of results for each round separately is highly advisable in order to make the evolving of consensus over the rounds transparent. This includes figures showing the average group response, changes between rounds, as well as any modifications of the survey instrument such as deletion, addition or modification of survey items based on previous rounds.	Figure 1<br>Tables S5-S7<br>Appendices 3-5
Discussion of limitations	14	Reporting should include a critical reflection of potential limitations and their impact of the resulting guidance.	Strengths and limitations
Adequacy of conclusions	15	The conclusions should adequately reflect the outcomes of the Delphi study with a view to the scope and applicability of the resulting practice guidance.	Recommendations
Publication and dissemination	16	The resulting guidance on good practice in palliative care should be clearly identifiable from the publication, including recommendations for transfer into practice and implementation. If the publication does not allow for a detailed presentation of either the resulting practice guidance or the methodological features of the applied Delphi technique, or both, reference to a more detailed presentation elsewhere should be made (e.g. availability of the full guideline from the authors or online; publication of a separate paper reporting on methodological details and particularities of the process (e.g. persistent disagreement and controversy on certain issues)). A dissemination plan should include endorsement of the guidance by professional associations and health care authorities to facilitate implementation.	Recommendations<br>Detailed report reviewed by the Medusa Advisory Board

[a]Junger S, Payne SA, Brine J, et al. Guidance on Conducting and REporting DElphi Studies (CREDES) in palliative care: recommendations based on a methodological systematic review. *Palliat Med* 2017; 31: 684-706. 2017/02/14. DOI: 10.1177/0269216317690685.

Table S3: p-values from Dunn-Bonferroni pairwise comparisons of the importance ratings of the 13 types of information using data from all Medusa users. An initial Friedman test was significant ($\chi^2(12) = 339.197$, $p<0.001$). A value of $p<0.05$ (shaded green) suggests a significant difference in importance rating, with the arrow indicating the information that was rated significantly more important.

	Compatibility	Incompatibility	Drug concentrations	Infusion fluids	How long compatible	How long for incompatibility	Temperatures	Light or darkness	Container material	Line or container	How compatibility measured	pH	Information source
Compatibility	-	1.000	1.000	1.000	0.055	←0.001	←<0.001	←0.001	←<0.001	←0.028	←<0.001	←<0.001	←<0.001
Incompatibility	-	-	0.761	1.000	←0.004	←<0.001	←<0.001	←<0.001	←<0.001	←0.002	←<0.001	←<0.001	←<0.001
Drug concentrations	-	-	-	1.000	1.000	1.000	←0.003	1.000	0.062	1.000	←<0.001	←<0.001	←<0.001
Infusion fluids	-	-	-	-	←0.014	←<0.001	←<0.001	←<0.001	←<0.001	←0.007	←<0.001	←<0.001	←<0.001
How long compatible	-	-	-	-	-	1.000	0.695	1.000	1.000	1.000	0.164	←0.003	0.133
How long for incompatibility	-	-	-	-	-	-	1.000	1.000	1.000	1.000	1.000	0.130	1.000
Temperatures	-	-	-	-	-	-	-	1.000	1.000	1.000	1.000	1.000	1.000
Light or darkness	-	-	-	-	-	-	-	-	1.000	1.000	1.000	0.126	1.000
Container material	-	-	-	-	-	-	-	-	-	1.000	1.000	1.000	1.000
Line or container	-	-	-	-	-	-	-	-	-	-	0.295	←0.007	0.241
How compatibility measured	-	-	-	-	-	-	-	-	-	-	-	1.000	1.000
pH	-	-	-	-	-	-	-	-	-	-	-	-	1.000

Table S4: the number of Delphi rounds to which participants contributed.

	Number who participated in one round	Number who participated in two rounds	Number who participated in three rounds
Nurses	2	9	5
Pharmacists	1	3	21

Table S5: participants' agreement with the consensus statements included in round 1 and consequent decision regarding inclusion of these statements in round 2.

	Statement	Round 1 – median agreement rating (IQR)			Round 1 outcome	Round 2 decision
		All participants (n=35)	Nurses (n=12)	Pharmacists (n=23)		
1	Only IV guides on Medusa for an AGREED LIST of relevant primary drugs should include compatibility information	2.0 (2.00)	2.0 (3.00)	2.0 (2.00)	No consensus	Remove, as alternative system (statements 2&3) accepted
2	All IV drug guides on Medusa for individual primary drugs given by CONTINUOUS INFUSION should include information on compatibility	6.0 (1.00)	6.0 (1.00)	6.0 (1.00)	Accepted	Include to measure stability of responses
3	All IV drug guides on Medusa for individual primary drugs given by SHORT INFUSION should include information on compatibility	5.0 (1.00)	5.0 (1.00)	5.0 (1.00)	Accepted	Include to measure stability of responses
4	All IV drug guides on Medusa for individual primary drugs given by INJECTION should include information on compatibility	4.0 (3.00)	4.5 (3.50)	3.0 (3.00)	No consensus	Include
5	Only secondary drugs from an AGREED LIST of relevant drugs should included	2.0 (2.00)	2.0 (2.50)	2.0 (2.00)	No consensus	Remove, as alternative system (statements 6&7) accepted
6	All secondary drugs given by CONTINUOUS INFUSION should be included	6.0 (1.00)	6.0 (1.00)	6.0 (1.00)	Accepted	Include to measure stability of responses
7	All secondary drugs given by SHORT INFUSION should be included	5.0 (1.00)	5.0 (1.00)	5.0 (0.00)	Accepted	Include to measure stability of responses
8	All secondary drugs given by INJECTION should be included	4.0 (2.00)	4.5 (2.50)	4.0 (2.00)	No consensus	Include
9	The Medusa compatibility section should include information on drug combinations that are known to be COMPATIBLE	6.0 (1.00)	6.0 (1.00)	5.0 (1.00)	Accepted	Remove as consensus reached
10	The Medusa compatibility section should include information on drug combinations that are known to be INCOMPATIBLE	6.0 (1.00)	6.0 (1.00)	6.0 (1.00)	Accepted	Remove as consensus reached
11	The Medusa compatibility section should list relevant drug combinations where compatibility is NOT KNOWN	3.0 (3.00)	5.0 (3.50)	3.0 (3.00)	No consensus	Include
12	The Medusa compatibility section should state that drug combinations are COMPATIBLE based on reports of visual inspection of the mixture for signs of incompatibility	4.0 (2.25)	5.0 (3.00)	4.0 (2.00)	No consensus	Include
13	The Medusa compatibility section should state that drug combinations are INCOMPATIBLE based on reports of visual inspection of the mixture for signs of incompatibility	5.0 (1.00)	5.0 (1.00)	5.0 (1.00)	Accepted	Remove as consensus reached
14	The Medusa compatibility section should state that drug combinations are COMPATIBLE based on data from scientific instruments that detect particles	5.0 (2.00)	5.0 (1.00)	5.0 (3.00)	No consensus	Include
15	The Medusa compatibility section should state that drug combinations are INCOMPATIBLE based on data from scientific instruments that detect particles	5.0 (1.25)	5.0 (1.00)	5.0 (2.00)	No consensus	Include
16	The Medusa compatibility section should state that drug combinations are COMPATIBLE based on chemical analysis	5.0 (2.00)	5.0 (1.50)	5.0 (2.00)	No consensus	Revise statement for clarity, based on free text responses
17	The Medusa compatibility section should state that drug combinations are INCOMPATIBLE based on chemical analysis	5.0 (1.00)	5.0 (1.00)	5.0 (2.00)	No consensus	Revise statement for clarity, based on free text responses
18	The Medusa compatibility section should only state that a pair of drugs are compatible when the supporting data relate to drug concentrations used in practice	5.0 (1.00)	4.5 (1.00)	5.0 (1.00)	Uncertain	Include
19	The Medusa compatibility section should state the concentrations at which two drugs are compatible	5.0 (2.00)	5.0 (1.75)	5.0 (2.00)	No consensus	Include
20	The Medusa compatibility section should only state that a pair of drugs are compatible when the supporting data relate to infusion solutions used in practice	5.0 (1.25)	5.0 (2.00)	5.0 (1.00)	No consensus	Revise statement for clarity, based on free text responses
21	The Medusa compatibility section should state the infusion solutions in which two drugs are compatible	5.0 (1.00)	5.0 (2.00)	5.0 (1.00)	No consensus	Revise statement for clarity, based on free text responses
22	The Medusa compatibility section should only state that a pair of drugs are compatible when this is over a time scale relevant to practice	5.0 (1.25)	4.0 (2.00)	5.0 (1.00)	No consensus	Include

23	The Medusa compatibility section should state for how long a pair of drugs are COMPATIBLE	5.0 (1.00)	4.5 (1.00)	5.0 (2.00)	No consensus	Include
24	The Medusa compatibility section should state how long it takes a pair of drugs to become INCOMPATIBLE	5.0 (2.00)	4.0 (2.00)	5.0 (2.00)	No consensus	Include
25	The Medusa compatibility section should only state that a pair of drugs are compatible when the supporting data relate to temperatures relevant to administration (not storage) in practice	4.0 (1.00)	5.0 (3.00)	4.0 (1.00)	No consensus	Include
26	The Medusa compatibility section should state the temperatures at which two drugs are compatible	4.0 (3.00)	4.0 (3.00)	4.0 (2.00)	No consensus	Include
27	The Medusa compatibility section should only state that a pair of drugs are compatible when the supporting data relate to administration in light (not protected from light)	4.0 (2.00)	5.0 (4.00)	3.0 (1.00)	No consensus	Include
28	The Medusa compatibility section should state whether two drugs are compatible in light or darkness	4.0 (1.00)	4.5 (1.75)	4.0 (1.00)	Uncertain	Split into two statements (darkness and light) based on free text responses
29	The Medusa compatibility section should only state that a pair of drugs are compatible when the supporting data relate to an IV container and/or IV line material used in practice	4.0 (3.00)	2.5 (3.00)	4.0 (2.00)	No consensus	Include
30	Where relevant, the Medusa compatibility section should state what the IV container and/or IV line containing a mixture of two drugs should be made from (e.g. glass, PVC)	5.0 (2.00)	5.0 (4.00)	5.0 (2.00)	No consensus	Revise statement for clarity, based on free text responses
31	The Medusa compatibility section should state that a pair of drugs are compatible when the supporting data relate to mixing in the same container (e.g. bag, syringe)	5.0 (2.00)	3.0 (3.00)	5.0 (2.00)	No consensus	Include
32	The Medusa compatibility section should state that a pair of drugs are compatible when the supporting data relate to mixing in the IV line	5.0 (0.25)	5.0 (1.00)	5.0 (1.00)	Accepted	Remove as consensus reached
33	The Medusa compatibility section should describe whether compatibility is for mixing in the IV line or in the same container (e.g. bag, syringe)	5.0 (1.00)	5.0 (0.00)	5.0 (1.00)	Accepted	Remove as consensus reached
34	The Medusa compatibility section should include the pH of the individual drugs. (NB: Medusa also provides this pH in a separate section).	4.0 (3.00)	4.0 (3.50)	4.0 (3.00)	No consensus	Revise statement for clarity, based on free text responses
35	The Medusa compatibility section should include details on where information came from e.g. references	5.0 (2.00)	5.0 (1.00)	5.0 (2.00)	No consensus	Include
36	The Medusa compatibility section should include information on how compatibility was measured e.g. visual inspection for cloudiness, chemical analysis etc.	5.0 (3.00)	4.0 (3.25)	5.0 (2.00)	No consensus	Revise statement for clarity, based on free text responses

IQR = interquartile range; IV = intravenous

Table S6: participants' agreement with the consensus statements included in round 2 and consequent decision regarding inclusion of these statements in round 3. Statements are presented in the order in which they were included in the online survey.

	Statement	Round 2 – median agreement rating (IQR)			Round 2 outcome	Round 3 decision
		All participants (n=35)	Nurses (n=12)	Pharmacists (n=23)		
2	All IV drug guides on Medusa for individual primary drugs given by CONTINUOUS INFUSION should include information on compatibility	6.0 (0.00)	6.0 (0.00)	6.0 (0.00)	Accepted	Remove as consensus reached in R1&2
3	All IV drug guides on Medusa for individual primary drugs given by SHORT INFUSION should include information on compatibility	5.0 (1.00)	6.0 (1.00)	5.0 (0.00)	Accepted	Remove as consensus reached in R1&2
4	All IV drug guides on Medusa for individual primary drugs given by INJECTION should include information on compatibility	4.0 (2.00)	4.5 (2.00)	4.0 (1.00)	No consensus	Revise statement for clarity, based on acceptance of statements 2&3
6	All secondary drugs given by CONTINUOUS INFUSION should be included	6.0 (0.00)	6.0 (0.75)	6.0 (0.00)	Accepted	Remove as consensus reached in R1&2
7	All secondary drugs given by SHORT INFUSION should be included	5.0 (1.00)	5.5 (1.00)	5.0 (0.00)	Accepted	Remove as consensus reached in R1&2
8	All secondary drugs given by INJECTION should be included	4.0 (2.00)	4.5 (3.50)	4.0 (1.00)	No consensus	Revise statement for clarity, based on acceptance of statements 6&7
11	The Medusa compatibility section should list relevant drug combinations where compatibility is NOT KNOWN	3.0 (3.00)	3.0 (3.50)	2.0 (3.00)	No consensus	Revise statement for clarity, based on free text responses
12	The Medusa compatibility section should state that drug combinations are COMPATIBLE based on reports of visual inspection of the mixture for signs of incompatibility	4.0 (3.00)	3.0 (3.00)	4.0 (3.00)	No consensus	Include
14	The Medusa compatibility section should state that drug combinations are COMPATIBLE based on data from scientific instruments that detect particles	5.0 (1.00)	5.0 (1.50)	5.0 (1.00)	Accepted	Include to measure stability of responses
15	The Medusa compatibility section should state that drug combinations are INCOMPATIBLE based on data from scientific instruments that detect particles	5.0 (1.00)	5.5 (1.00)	5.0 (1.00)	Accepted	Include to measure stability of responses
16 (R2)	The Medusa compatibility section should state that drug combinations are COMPATIBLE based on chemical analysis that can detect degradation of drugs that does NOT result in cloudiness or particle formation	5.0 (1.00)	5.5 (1.75)	5.0 (1.00)	Accepted	Remove as consensus reached
17 (R2)	The Medusa compatibility section should state that drug combinations are INCOMPATIBLE based on chemical analysis that can detect degradation of drugs that does NOT result in cloudiness or particle formation	5.0 (1.00)	6.0 (1.00)	5.0 (1.00)	Accepted	Remove as consensus reached
18	The Medusa compatibility section should only state that a pair of drugs are compatible when the supporting data relate to drug concentrations used in practice	5.0 (1.00)	4.0 (2.50)	5.0 (1.00)	No consensus	Include
19	The Medusa compatibility section should state the concentrations at which two drugs are compatible	5.0 (2.00)	5.0 (1.50)	5.0 (2.00)	No consensus	Include
20 (R2)	The Medusa compatibility section should only state that a pair of drugs are compatible when the supporting data relate to infusion solutions (diluents) used in practice	5.0 (1.00)	5.0 (2.50)	5.0 (0.00)	No consensus	Include
21 (R2)	The Medusa compatibility section should state the infusion solutions (diluents) in which two drugs are compatible	5.0 (1.00)	6.0 (1.00)	5.0 (1.00)	Accepted	Remove as consensus reached
22	The Medusa compatibility section should only state that a pair of drugs are compatible when this is over a time scale relevant to practice	5.0 (0.00)	5.0 (1.75)	5.0 (0.00)	Accepted	Remove as consensus reached
23	The Medusa compatibility section should state for how long a pair of drugs are COMPATIBLE	5.0 (1.00)	4.5 (3.50)	5.0 (1.00)	No consensus	Include

24	The Medusa compatibility section should state how long it takes a pair of drugs to become INCOMPATIBLE	5.0 (2.00)	5.0 (2.00)	5.0 (4.00)	No consensus	Include
25	The Medusa compatibility section should only state that a pair of drugs are compatible when the supporting data relate to temperatures relevant to administration (not storage) in practice	5.0 (1.00)	5.0 (1.00)	5.0 (1.00)	Accepted	Remove as consensus reached
26	The Medusa compatibility section should state the temperatures at which two drugs are compatible	4.0 (2.00)	4.0 (2.00)	4.0 (0.00)	No consensus	Include
27	The Medusa compatibility section should only state that a pair of drugs are compatible when the supporting data relate to administration in light (not protected from light)	4.0 (2.00)	4.5 (2.00)	4.0 (1.25)	No consensus	Include
28.1	The Medusa compatibility section should state when two drugs are compatible in light	4.0 (2.00)	4.5 (2.00)	4.0 (2.00)	No consensus	Include
28.2	The Medusa compatibility section should state when two drugs are only compatible in darkness	5.0 (3.00)	3.5 (3.00)	5.0 (2.00)	No consensus	Include
29	The Medusa compatibility section should only state that a pair of drugs are compatible when the supporting data relate to an IV container and/or IV line material used in practice	5.0 (2.00)	4.5 (2.75)	5.0 (1.00)	No consensus	Include
30 (R2)	If there are specific requirements, the Medusa compatibility section should state what the IV container and/or IV line containing a mixture of two drugs should be made from (e.g. glass, PVC)	5.0 (1.00)	5.0 (1.75)	6.0 (1.00)	Accepted	Remove as consensus reached
31	The Medusa compatibility section should state that a pair of drugs are compatible when the supporting data relate to mixing in the same container (e.g. bag, syringe)	5.0 (0.00)	5.0 (0.00)	5.0 (0.00)	Accepted	Remove as consensus reached
36 (R2)	The Medusa compatibility section should include information on how the compatibility data on which it is based was obtained, e.g. visual inspection for cloudiness, chemical analysis etc.	5.0 (2.00)	5.0 (2.00)	5.0 (2.00)	No consensus	Include
34 (R2)	The Medusa compatibility section should include the pH of the individual drugs in ADDITION to it already being provided in the separate pH section.	4.0 (2.00)	4.0 (2.75)	3.0 (2.00)	No consensus	Include
35	The Medusa compatibility section should include details on where information came from e.g. references	5.0 (3.00)	4.5 (2.75)	5.0 (3.00)	No consensus	Include

IQR = interquartile range; IV = intravenous, R2 = round 2 wording

Table S7: participants' agreement with the consensus statements included in round 3. Statements are presented in the order in which they were included in the online survey.

	Statement	Round 3 – median agreement rating (IQR)			Round 3 outcome
		All participants (n=35)	Nurses (n=11)	Pharmacists (n=24)	
4 (R3)	All IV guides on Medusa for drugs given by INJECTION ONLY should include information on their compatibility with other drugs	4.0 (2.00)	6.0 (2.00)	4.0 (2.00)	No consensus
8 (R3)	All IV guides on Medusa for drugs given by CONTINUOUS OR SHORT INFUSION should include information on their compatibility with drugs given by INJECTION ONLY	4.0 (1.00)	6.0 (2.00)	4.0 (1.00)	No consensus
11 (R3)	The Medusa compatibility section should list COMMON, IMPORTANT drug combinations where compatibility is NOT KNOWN	5.0 (3.00)	4.0 (3.00)	5.0 (3.00)	No consensus
37*	In ADDITION to 'Y-site' compatibility information, the Medusa compatibility section should include information on the mixing of drugs in the SAME CONTAINER (e.g. infusion bag, syringe)	4.5 (3.00)	5.0 (3.00)	4.0 (3.00)	No consensus
12	The Medusa compatibility section should state that drug combinations are COMPATIBLE based on reports of visual inspection of the mixture for signs of incompatibility	5.0 (3.00)	5.0 (3.00)	5.0 (3.00)	No consensus
14	The Medusa compatibility section should state that drug combinations are COMPATIBLE based on data from scientific instruments that detect particles	5.0 (0.00)	5.0 (1.00)	5.0 (0.00)	Accepted
15	The Medusa compatibility section should state that drug combinations are INCOMPATIBLE based on data from scientific instruments that detect particles	5.0 (1.00)	5.0 (1.00)	5.0 (1.00)	Accepted
18	The Medusa compatibility section should only state that a pair of drugs are compatible when the supporting data relate to drug concentrations used in practice	5.0 (0.00)	5.0 (1.00)	5.0 (0.00)	Accepted
19	The Medusa compatibility section should state the concentrations at which two drugs are compatible	5.0 (0.00)	5.0 (0.25)	5.0 (1.00)	Accepted
20 (R2)	The Medusa compatibility section should only state that a pair of drugs are compatible when the supporting data relate to infusion solutions (diluents) used in practice	5.0 (0.00)	5.0 (1.00)	5.0 (0.00)	Accepted
23	The Medusa compatibility section should state for how long a pair of drugs are COMPATIBLE	5.0 (1.00)	5.0 (1.00)	5.0 (1.00)	Accepted
24	The Medusa compatibility section should state how long it takes a pair of drugs to become INCOMPATIBLE	5.0 (1.00)	5.0 (3.00)	5.0 (1.00)	No consensus
26	The Medusa compatibility section should state the temperatures at which two drugs are compatible	4.0 (1.00)	4.0 (2.00)	4.0 (1.00)	No consensus
27	The Medusa compatibility section should only state that a pair of drugs are compatible when the supporting data relate to administration in light (not protected from light)	4.0 (2.00)	5.0 (1.00)	4.0 (2.00)	No consensus
28.1	The Medusa compatibility section should state when two drugs are compatible in light	5.0 (2.00)	5.0 (1.00)	4.0 (2.00)	No consensus
28.2	The Medusa compatibility section should state when two drugs are only compatible in darkness	5.0 (1.00)	5.0 (3.00)	5.0 (1.00)	No consensus
29	The Medusa compatibility section should only state that a pair of drugs are compatible when the supporting data relate to an IV container and/or IV line material used in practice	5.0 (0.00)	5.0 (2.00)	5.0 (0.00)	No consensus
36 (R2)	The Medusa compatibility section should include information on how the compatibility data on which it is based was obtained, e.g. visual inspection for cloudiness, chemical analysis etc.	5.0 (1.00)	5.0 (2.00)	5.0 (1.00)	No consensus
34 (R2)	The Medusa compatibility section should include the pH of the individual drugs in ADDITION to it already being provided in the separate pH section	3.0 (2.00)	4.0 (4.00)	2.5 (2.00)	No consensus
35	The Medusa compatibility section should include details on where information came from e.g. references	6.0 (1.00)	5.0 (3.00)	6.0 (1.00)	No consensus

*New statement added for round 3 in response to specific request from Medusa editorial team.
IQR = interquartile range; IV = intravenous; R2 = round 2 wording; R3 = round 3 wording

Appendix 1: Drugs for which Medusa currently provides compatibility information:

The following drugs are listed in the Medusa writing guide, although in practice information is provided for a wider range:

- All drugs in the ‘Standard Medication Concentrations in Adult Critical Care’ list
- Drugs listed on the ‘Thames Valley Y-Site Intravenous Drug Compatibility Chart’.
- Anti-infectives
- Analgesics
- Medicines frequently prescribed post-op (e.g. for nausea and vomiting) Immunosuppressants used post transplants (e.g. cyclosporin, tacrolimus)
- Steroids
- Intravenous infusion fluids

Compatibility of IV drugs survey

Page 1: Introduction

If you use Medusa (the NHS Injectable Medicines Guide) whether as a nurse, a pharmacist, a doctor or another profession, please take 5-10 minutes to complete this 11-question anonymous survey about your experiences of IV drug compatibility. It has been organised by the team that manages Medusa, as part of a service evaluation registered at Imperial College Healthcare NHS Trust (number 863).

Only the Medusa team will have access to the information that you provide. Your responses will be stored anonymously, so it will not be possible to identify you from them. They will be treated as confidential and kept on a password protected file. Recorded data will be kept for at least 10 years. A summary of all responses may be published in reports about this project, but this will not include any identifiable information.

You are free to stop completing the survey at any time, without giving a reason. After you have clicked the finish button, it is not possible to withdraw your data as it will be anonymous.

What do I do now?
If you would like to complete this survey, please answer the question below and click the 'Next' button. Please note that **the survey is not saved automatically**. Please answer as many questions as you want to and then move to the last page and **click the finish button**, otherwise your answers will not be saved.

If you wish to save your responses and finish the survey later, please click on the 'Finish later' option at the bottom of each page. This will take you to a new page with a link you can save to come back to your partly completed survey.

Thank you for your help.

1. If you have read the information above and agree to take part with the understanding that the data (including any personal data) you submit will be processed accordingly, please select the box below to get started. ✱ *Required*

1 / 12

○ I agree to take part

Page 2: Use of IV drugs via the same IV line or lumen

This page asks about your experience of the use of IV drugs via the same IV line or lumen. By this we mean administering:

- Two or more IV drugs
- At the same time
- Via the same IV line or lumen, so they mix before entering the bloodstream
- This usually requires use of a 'Y-site' connector, such as a double- or triple-lumen needle-free extension set (e.g. Octopus) or a 'three way tap'.

Please answer the questions based on your own professional perspective, whether that's prescribing, preparing, administering or advising on IV drug administration.

2. Over the past year, how often have you been involved with the use of IV drugs via the same IV line or lumen (following the definition above)? *Please chose the answer that most closely matches your experience.*

- Every day
- Every week
- Every month
- Less than once a month
- Never

3. Over the past year, which of the following drugs have you administered or seen administered via the same IV line or lumen (following the definition above)?

- Antibiotics
- Antifungals
- Antivirals
- Analgesics e.g. paracetamol, morphine
- Steroids e.g. hydrocortisone

- Immunosuppressants e.g. ciclosporin, tacrolimus
- IV fluids e.g. sodium chloride, glucose, Hartmann's
- Electrolytes e.g. potassium chloride, magnesium sulphate
- Cardioactive drugs e.g. amiodarone, isosorbide dinitrate
- Diuretics e.g. furosemide
- Vasopressors and inotropes, e.g. adrenaline, noradrenaline, dobutamine, isoprenaline
- Acid suppressants e.g. omeprazole, ranitidine
- Sedatives e.g. midazolam, propofol, clonidine
- Muscle relaxants e.g. atracurium, vecuronium
- Antiepileptics e.g. phenobarbital, phenytoin, sodium valproate
- Anti-emetics e.g. ondansetron, cyclizine
- Insulin
- Heparin
- Tranexamic acid
- Acetylcysteine
- Aminophylline
- Vasopressin/argipressin
- Other

3.a. If you selected Other, please specify:

Page 3: Information about the compatibility of IV drugs in solution

This section asks about the information you might need when you're involved with the use of IV drugs via the same IV line or lumen. By this we mean administering:

- Two or more IV drugs
- At the same time
- Via the same IV line or lumen, so they mix before entering the bloodstream
- This usually requires use of a 'Y-site' connector, such as a double- or triple -lumen needle-free extension set (e.g. Octopus) or a ‘three way tap’.

Please answer the questions based on your own professional perspective, whether that's prescribing, preparing, administering or advising on IV drug administration.

4. How important is the following information when deciding how to manage a patient who may need IV drugs administered via the same IV line or lumen (following the definition above)?

Please don't select more than 1 answer(s) per row.

	Very unimportant	Unimportant	Neither important nor unimportant	Important	Very important
Which drugs are COMPATIBLE with each other	☐	☐	☐	☐	☐
Which drugs are INCOMPATIBLE with each other	☐	☐	☐	☐	☐

5. How important is the following additional information about the compatibility or incompatibility of drugs? *When the options below refer to 'compatibility', this also includes 'incompatibility'.*

Please don't select more than 1 answer(s) per row.

	Very unimportant	Unimportant	Neither important nor unimportant	Important	Very important
Drug concentrations where drugs are compatible	☐	☐	☐	☐	☐
Infusion fluids in which drugs are compatible	☐	☐	☐	☐	☐
For how long drugs are compatible	☐	☐	☐	☐	☐
How long it takes drugs to become incompatible	☐	☐	☐	☐	☐
Temperatures at which drugs are compatible	☐	☐	☐	☐	☐
Whether the drugs are compatible in light or darkness	☐	☐	☐	☐	☐
What the IV container (bag, syringe) and IV line should be made from (e.g. glass, PVC)	☐	☐	☐	☐	☐

Whether the information is for mixing in the IV line or in the same container (e.g. bag, syringe)	☐	☐	☐	☐	☐
How compatibility was measured e.g. visual inspection for cloudiness, chemical analysis etc.	☐	☐	☐	☐	☐
The pH of the drugs	☐	☐	☐	☐	☐
Where the information came from e.g. references	☐	☐	☐	☐	☐

You're about halfway through the survey. Please keep going as your answers are important to the project.

Remember to answer as many questions as you want to and then move to the last page and **click the finish button**, otherwise your answers will not be saved.

Page 4: Medusa

6. Over the past year, how often have you used Medusa (the NHS Injectable Medicines Guide) for **any** information about IV drugs? *Please chose the answer that most closely matches your experience.*

- Every day
- Every week
- Every month
- Less than once a month
- Never

7. Over the past year, how often have you used the **'Compatibility' section** found in each IV drug guide on Medusa (the NHS Injectable Medicines Guide)? *Please chose the answer that most closely matches your experience.*

- Every day
- Every week
- Every month
- Less than once a month
- Never
- I didn't know it existed

Just two more pages to go!

Page 5: Medusa compatibility section

8. How much do you agree with the following statements about the Medusa **'Compatibility'** section?

Please don't select more than 1 answer(s) per row.

	Strongly disagree	Disagree	Neither agree nor disagree	Agree	Strongly agree	I haven't seen it
It is easy to find	☐	☐	☐	☐	☐	☐
It is easy to understand	☐	☐	☐	☐	☐	☐
It is easy to use	☐	☐	☐	☐	☐	☐
It is useful	☐	☐	☐	☐	☐	☐
It is intended for someone doing a job like me	☐	☐	☐	☐	☐	☐

9. How do you think the Medusa **'Compatibility'** section could be improved? *You may want to think about the areas described in the question above, or anything else.*

Just one more page to go!

Page 6: About you

This is the last page! There are three questions to tell us a little bit about you, but you don't have to answer them if you'd rather not. Whether you answer them or not, please **click the 'Finish' button** below to save your previous answers.

10. Which profession do you belong to?

- Nurse or midwife
- Doctor
- Pharmacy professional
- Allied health professional
- Other

10.a. If you selected Other, please specify:

11. How many years have you been qualified?

Please enter a number.

12. In which clinical areas have you worked over the past year?

	Adults	Children
Ambulance service	☐	☐
Emergency department	☐	☐

10 / 12

Inpatient wards	☐	☐
Critical or intensive care	☐	☐
Theatres and/or recovery	☐	☐
Ambulatory or day care	☐	☐
Outpatients	☐	☐
Hospital at home	☐	☐
Community	☐	☐
Mental health	☐	☐
Other	☐	☐

12.a. If you selected 'other' above, please give more details.

Page 7: Thank you

Thank you for your responses, which will be very useful for improving Medusa.

Drug compatibility information in Medusa - round 1 survey

Page 1: Introduction

This is a 20 minute survey about your opinions on the information on IV drug compatibility that should be provided in Medusa, the NHS Injectable Medicines Guide.

This is the first of up to three related surveys that you will be invited to complete. The second and third surveys will show you an anonymous summary of responses to the previous survey. They will then repeat some or all of the questions to find out if this changes people's views. In this way, it may be possible to develop agreement about what information on IV drug compatibility should be provided in Medusa. As you can appreciate from this, your responses to all three surveys are therefore important and your contribution of valuable time to do this is much appreciated.

This service evaluation project has been organised by the team that manages Medusa and is registered at Imperial College Healthcare NHS Trust (number 863). Only the Medusa team will have access to the information that you provide and it will not be shared with anyone else in an identifiable way. They will be treated as confidential and kept password protected. Once the three surveys are complete, the data will be anonymised and kept for at least 10 years. A summary of all responses may be published in reports about this project, but this will not include any identifiable information.

You are free to stop completing the survey at any time, without giving a reason. After you have clicked the finish button, it may not be possible to withdraw your data, as it will soon be used to prepare the next survey.

What do I do now?
If you would like to complete this survey, please answer the question below and click the 'Next' button. Please note that **the survey is not saved automatically**. Please answer as many questions as you want to and then move to the last page and **click the finish button**, otherwise your answers will not be saved.

If you wish to save your responses and finish the survey later, please click on the 'Finish later' option at the bottom of each page. This will take you to a new page with a link you can save to come back to your partly completed survey.

Thank you for your help.

1. If you have read the information above and agree to take part with the understanding that the data (including any personal data) you submit will be processed accordingly, please select the box below to get started. ✱ *Required*

○ I agree to take part

Page 2: Drugs for which compatibility information is provided

This survey is about the information provided by Medusa to support staff administering IV drugs via the same IV line or lumen. By this we mean administering:

- Two or more IV drugs
- At the same time
- Via the same IV line or lumen, so they mix before entering the bloodstream
- This usually requires use of a 'Y-site' connector, such as a double- or triple -lumen needle-free extension set (e.g. Octopus) or a ‘three way tap’.

This survey usually uses the word **'compatibility'** to mean information on whether two drugs are **stable or not** when mixed in the same IV line or lumen.

The next two questions are about the drug combinations for which compatibility information should be given, in order to provide a balance betwen comprehensiveness and information overload. You can think of this in two stages:

1. Which drugs should have compatibility information included in their IV guide on Medusa? This survey refers to these drugs as the '**primary drug**'. *For example, you might think it's important to provide this information for all drugs, or you might think it's only needed for certain drugs or those given by continuous infusion, etc.*
2. Once it has been decided to include compatibility information in the Medusa guide for a particular primary drug, we then need to decide which drug combinations with '**secondary drugs**' should be included. *For example, you might think it's important to provide all the available compatibility information, or you might think that information is only needed for compatibility with secondary drugs given by continuous or short infusion, etc.*

The difference between primary and secondary drugs is illustrated in this screenshot from the Medusa monograph for fentanyl:

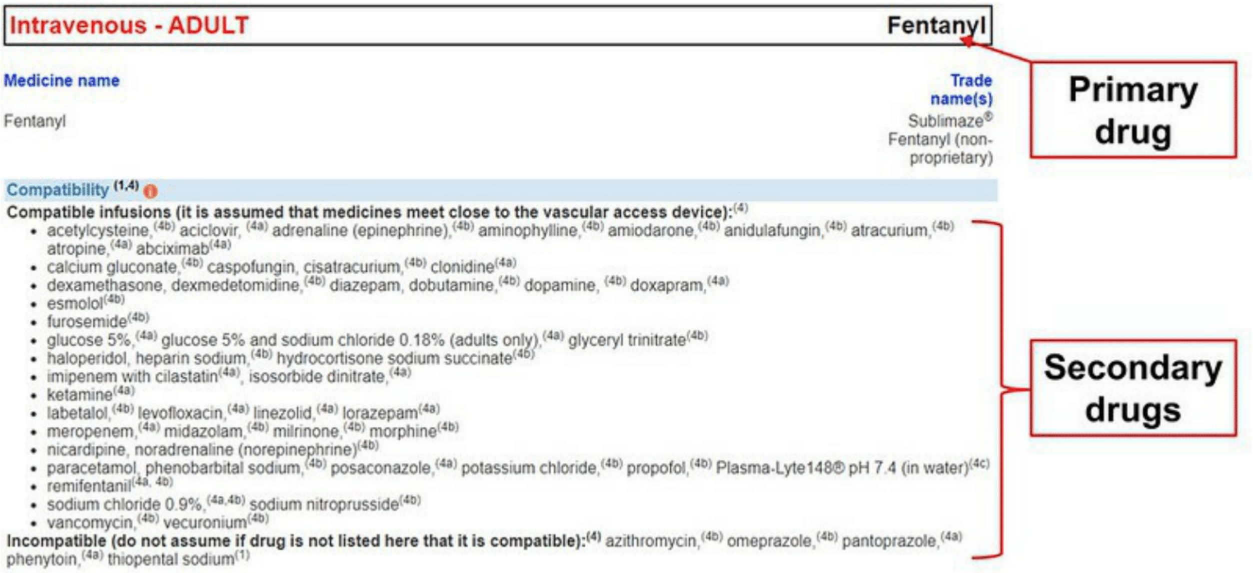
Intravenous - ADULT **Fentanyl**

Medicine name

Fentanyl

Trade name(s)

Sublimaze®
Fentanyl (non-proprietary)

Compatibility [1,4]

Compatible infusions (it is assumed that medicines meet close to the vascular access device):[4]

- acetylcysteine,[4b] aciclovir, [4a] adrenaline (epinephrine),[4b] aminophylline,[4b] amiodarone,[4b] anidulafungin,[4b] atracurium,[4b] atropine,[4a] abciximab[4a]
- calcium gluconate,[4b] caspofungin, cisatracurium,[4b] clonidine[4a]
- dexamethasone, dexmedetomidine,[4b] diazepam, dobutamine,[4b] dopamine, [4b] doxapram,[4a]
- esmolol[4b]
- furosemide[4b]
- glucose 5%,[4a] glucose 5% and sodium chloride 0.18% (adults only),[4a] glyceryl trinitrate[4b]
- haloperidol, heparin sodium,[4b] hydrocortisone sodium succinate[4b]
- imipenem with cilastatin[4a], isosorbide dinitrate,[4a]
- ketamine[4a]
- labetalol,[4b] levofloxacin,[4a] linezolid,[4a] lorazepam[4a]
- meropenem,[4a] midazolam,[4b] milrinone,[4b] morphine[4b]
- nicardipine, noradrenaline (norepinephrine)[4b]
- paracetamol, phenobarbital sodium,[4b] posaconazole,[4a] potassium chloride,[4b] propofol,[4b] Plasma-Lyte148® pH 7.4 (in water)[4c]
- remifentanil[4a, 4b]
- sodium chloride 0.9%,[4a,4b] sodium nitroprusside[4b]
- vancomycin,[4b] vecuronium[4b]

Incompatible (do not assume if drug is not listed here that it is compatible):[4] azithromycin,[4b] omeprazole,[4b] pantoprazole,[4a] phenytoin,[4a] thiopental sodium[1]

However, when considered from the point of view of the Medusa guide for adrenaline (now the primary drug), fentanyl becomes one of the secondary drugs:

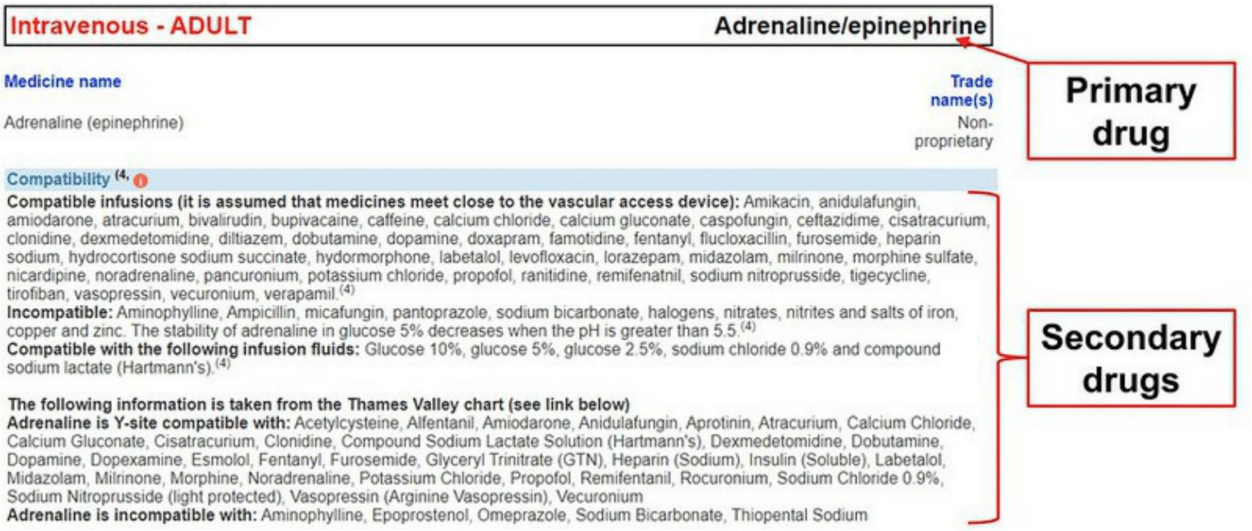

Intravenous - ADULT **Adrenaline/epinephrine**

Medicine name Adrenaline (epinephrine)

Trade name(s) Non-proprietary

Compatibility (4,

Compatible infusions (it is assumed that medicines meet close to the vascular access device): Amikacin, anidulafungin, amiodarone, atracurium, bivalirudin, bupivacaine, caffeine, calcium chloride, calcium gluconate, caspofungin, ceftazidime, cisatracurium, clonidine, dexmedetomidine, diltiazem, dobutamine, dopamine, doxapram, famotidine, fentanyl, flucloxacillin, furosemide, heparin sodium, hydrocortisone sodium succinate, hydormorphone, labetalol, levofloxacin, lorazepam, midazolam, milrinone, morphine sulfate, nicardipine, noradrenaline, pancuronium, potassium chloride, propofol, ranitidine, remifenatnil, sodium nitroprusside, tigecycline, tirofiban, vasopressin, vecuronium, verapamil.(4)
Incompatible: Aminophylline, Ampicillin, micafungin, pantoprazole, sodium bicarbonate, halogens, nitrates, nitrites and salts of iron, copper and zinc. The stability of adrenaline in glucose 5% decreases when the pH is greater than 5.5.(4)
Compatible with the following infusion fluids: Glucose 10%, glucose 5%, glucose 2.5%, sodium chloride 0.9% and compound sodium lactate (Hartmann's).(4)

The following information is taken from the Thames Valley chart (see link below)
Adrenaline is Y-site compatible with: Acetylcysteine, Alfentanil, Amiodarone, Anidulafungin, Aprotinin, Atracurium, Calcium Chloride, Calcium Gluconate, Cisatracurium, Clonidine, Compound Sodium Lactate Solution (Hartmann's), Dexmedetomidine, Dobutamine, Dopamine, Dopexamine, Esmolol, Fentanyl, Furosemide, Glyceryl Trinitrate (GTN), Heparin (Sodium), Insulin (Soluble), Labetalol, Midazolam, Milrinone, Morphine, Noradrenaline, Potassium Chloride, Propofol, Remifentanil, Rocuronium, Sodium Chloride 0.9%, Sodium Nitroprusside (light protected), Vasopressin (Arginine Vasopressin), Vecuronium
Adrenaline is incompatible with: Aminophylline, Epoprostenol, Omeprazole, Sodium Bicarbonate, Thiopental Sodium

2. How strongly do you agree or disagree with the following statements about the choice of the **primary drugs** for which compatibility information is provided? *If you think that compatibility information should be provided for **all** drugs, then choose the relevant agree option for the continuous infusion AND short infusion AND injection options above, as this will then cover all drugs. Note that Medusa will NOT provide compatibility information where this will always be inappropriate, e.g. parenteral nutrition.*

Please don't select more than 1 answer(s) per row.

	Strongly disagree	Disagree	Weakly disagree	Weakly agree	Agree	Strongly agree
Only IV guides on Medusa for an AGREED LIST of relevant primary drugs should include compatibility information	☐	☐	☐	☐	☐	☐
All IV drug guides on Medusa for individual primary drugs given by CONTINUOUS INFUSION should include information on compatibility	☐	☐	☐	☐	☐	☐

All IV drug guides on Medusa for individual primary drugs given by SHORT INFUSION should include information on compatibility	☐	☐	☐	☐	☐	☐
All IV drug guides on Medusa for individual primary drugs given by INJECTION should include information on compatibility	☐	☐	☐	☐	☐	☐

2.a. If you want to, please add a few words to explain why this is your opinion.

2.b. Do you think that the **primary drugs** for which compatibility information is included should be selected in a different way? If so, please explain here.

The next page will ask about your opinions on the secondary drugs to include in the compatibility section of Medusa for selected primary drugs.

Remember to answer as many questions as you want to and then move to the last page and **click the finish button**

Page 3: Drugs for which compatibility information is provided

3. How strongly do you agree or disagree with the following statements about the choice of **secondary drugs** to include in the compatibility section of Medusa for selected primary drugs? *If you think that information should be provided for **all** secondary drugs, then choose the relevant agree option for the continuous infusion AND short infusion AND injection options above, as this will then cover all drugs. Note that Medusa will NOT provide compatibility information where this will always be inappropriate, e.g. parenteral nutrition.*

Please don't select more than 1 answer(s) per row.

	Strongly disagree	Disagree	Weakly disagree	Weakly agree	Agree	Strongly agree
Only secondary drugs from an AGREED LIST of relevant drugs should included	☐	☐	☐	☐	☐	☐
All secondary drugs given by CONTINUOUS INFUSION should be included	☐	☐	☐	☐	☐	☐
All secondary drugs given by SHORT INFUSION should be included	☐	☐	☐	☐	☐	☐
All secondary drugs given by INJECTION should be included	☐	☐	☐	☐	☐	☐

3.a. If you want to, please add a few words to explain why this is your opinion.

3.b. Do you think that the **secondary drugs** for which compatibility information is given should be selected in a different way? If so, please explain here.

Page 4: Information in the Medusa compatibility section

After agreeing the drug combinations that should be included (i.e. the previous two questions), it's important to decide what information about these combinations should be provided and how strong the supporting data need to be. Once again, a balance betwen comprehensiveness and information overload is needed. The next two questions are about this issue.

Remember to answer as many questions as you want to and then move to the last page and **click the finish button**, otherwise your answers will not be saved.

4. How strongly do you agree or disagree with the following statements?

Please don't select more than 1 answer(s) per row.

	Strongly disagree	Disagree	Weakly disagree	Weakly agree	Agree	Strongly agree
The Medusa compatibility section should include information on drug combinations that are known to be COMPATIBLE	☐	☐	☐	☐	☐	☐
The Medusa compatibility section should include information on drug combinations that are known to be INCOMPATIBLE	☐	☐	☐	☐	☐	☐
The Medusa compatibility section should list relevant drug combinations where compatibility is NOT KNOWN	☐	☐	☐	☐	☐	☐

4.a. If you want to, please add a few words to explain why this is your opinion.

5. How strongly do you agree or disagree with the following statements?

Please don't select more than 1 answer(s) per row.

	Strongly disagree	Disagree	Weakly disagree	Weakly agree	Agree	Strongly agree

The Medusa compatibility section should state that drug combinations are COMPATIBLE based on reports of visual inspection of the mixture for signs of incompatibility	☐	☐	☐	☐	☐	☐
The Medusa compatibility section should state that drug combinations are INCOMPATIBLE based on reports of visual inspection of the mixture for signs of incompatibility	☐	☐	☐	☐	☐	☐
The Medusa compatibility section should state that drug combinations are COMPATIBLE based on data from scientific instruments that detect particles	☐	☐	☐	☐	☐	☐
The Medusa compatibility section should state that drug combinations are INCOMPATIBLE based on data from scientific instruments that detect particles	☐	☐	☐	☐	☐	☐
The Medusa compatibility section should state that drug combinations are COMPATIBLE based on chemical analysis	☐	☐	☐	☐	☐	☐
The Medusa compatibility section should state that drug combinations are INCOMPATIBLE based on chemical analysis	☐	☐	☐	☐	☐	☐

5.a. If you want to, please add a few words to explain why this is your opinion.

Page 5: Additional information in the Medusa compatibility section

Finally, it's important to decide what additional details should be provided about drug combinations. Of course, a balance between comprehensiveness and information overload is needed. The last section of the survey (11 questions on 6 pages) is about this issue.

Remember to answer as many questions as you want to and then move to the last page and **click the finish button**, otherwise your answers will not be saved.

6. How strongly do you agree or disagree with the following statements?

Please don't select more than 1 answer(s) per row.

	Strongly disagree	Disagree	Weakly disagree	Weakly agree	Agree	Strongly agree
The Medusa compatibility section should only state that a pair of drugs are compatible when the supporting data relate to drug concentrations used in practice	☐	☐	☐	☐	☐	☐
The Medusa compatibility section should state the concentrations at which two drugs are compatible	☐	☐	☐	☐	☐	☐

6.a. If you want to, please add a few words to explain why this is your opinion.

7. How strongly do you agree or disagree with the following statements?

Please don't select more than 1 answer(s) per row.

	Strongly disagree	Disagree	Weakly disagree	Weakly agree	Agree	Strongly agree
The Medusa compatibility section should only state that a pair of drugs are compatible when the supporting data relate to infusion solutions used in practice	☐	☐	☐	☐	☐	☐

The Medusa compatibility section should state the infusion solutions in which two drugs are compatible	☐	☐	☐	☐	☐	☐

7.a. If you want to, please add a few words to explain why this is your opinion.

Page 6: Additional information in the Medusa compatibility section

You're about halfway through the survey. Please keep going as your answers are important to the project.

Remember to answer as many questions as you want to and then move to the last page and **click the finish button**, otherwise your answers will not be saved.

8. How strongly do you agree or disagree with the following statements?

Please don't select more than 1 answer(s) per row.

	Strongly disagree	Disagree	Weakly disagree	Weakly agree	Agree	Strongly agree
The Medusa compatibility section should only state that a pair of drugs are compatible when this is over a time scale relevant to practice	☐	☐	☐	☐	☐	☐
The Medusa compatibility section should state for how long a pair of drugs are COMPATIBLE	☐	☐	☐	☐	☐	☐
The Medusa compatibility section should state how long it takes a pair of drugs to become INCOMPATIBLE	☐	☐	☐	☐	☐	☐

8.a. If you want to, please add a few words to explain why this is your opinion.

Page 7: Additional information in the Medusa compatibility section

9. How strongly do you agree or disagree with the following statements?

Please don't select more than 1 answer(s) per row.

	Strongly disagree	Disagree	Weakly disagree	Weakly agree	Agree	Strongly agree
The Medusa compatibility section should only state that a pair of drugs are compatible when the supporting data relate to temperatures relevant to administration (not storage) in practice	☐	☐	☐	☐	☐	☐
The Medusa compatibility section should state the temperatures at which two drugs are compatible	☐	☐	☐	☐	☐	☐

9.a. If you want to, please add a few words to explain why this is your opinion.

10. How strongly do you agree or disagree with the following statements?

Please don't select more than 1 answer(s) per row.

	Strongly disagree	Disagree	Weakly disagree	Weakly agree	Agree	Strongly agree
The Medusa compatibility section should only state that a pair of drugs are compatible when the supporting data relate to administration in light (not protected from light)	☐	☐	☐	☐	☐	☐
The Medusa compatibility section should state whether two drugs are compatible in light or darkness	☐	☐	☐	☐	☐	☐

10.a. If you want to, please add a few words to explain why this is your opinion.

Page 8: Additional information in the Medusa compatibility section

You're nearly at the end of the survey. Please keep going as your answers are important to the project.

Remember to answer as many questions as you want to and then move to the last page and **click the finish button**, otherwise your answers will not be saved.

11. How strongly do you agree or disagree with the following statements?

Please don't select more than 1 answer(s) per row.

	Strongly disagree	Disagree	Weakly disagree	Weakly agree	Agree	Strongly agree
The Medusa compatibility section should only state that a pair of drugs are compatible when the supporting data relate to an IV container and/or IV line material used in practice	☐	☐	☐	☐	☐	☐
Where relevant, the Medusa compatibility section should state what the IV container and/or IV line containing a mixture of two drugs should be made from (e.g. glass, PVC)	☐	☐	☐	☐	☐	☐

11.a. If you want to, please add a few words to explain why this is your opinion.

12. How strongly do you agree or disagree with the following statements?

Please don't select more than 1 answer(s) per row.

	Strongly disagree	Disagree	Weakly disagree	Weakly agree	Agree	Strongly agree

The Medusa compatibility section should state that a pair of drugs are compatible when the supporting data relate to mixing in the same container (e.g. bag, syringe)	☐	☐	☐	☐	☐	☐
The Medusa compatibility section should state that a pair of drugs are compatible when the supporting data relate to mixing in the IV line	☐	☐	☐	☐	☐	☐
The Medusa compatibility section should describe whether compatibility is for mixing in the IV line or in the same container (e.g. bag, syringe)	☐	☐	☐	☐	☐	☐

12.a. If you want to, please add a few words to explain why this is your opinion.

Page 9: Additional information in the Medusa compatibility section

13. How strongly do you agree or disagree with the following statement?

Please don't select more than 1 answer(s) per row.

	Strongly disagree	Disagree	Weakly disagree	Weakly agree	Agree	Strongly agree
The Medusa compatibility section should include information on how compatibility was measured e.g. visual inspection for cloudiness, chemical analysis etc.	☐	☐	☐	☐	☐	☐

13.a. If you want to, please add a few words to explain why this is your opinion.

14. How strongly do you agree or disagree with the following statement?

Please don't select more than 1 answer(s) per row.

	Strongly disagree	Disagree	Weakly disagree	Weakly agree	Agree	Strongly agree
The Medusa compatibility section should include the pH of the individual drugs. (NB: Medusa also provides this pH in a separate section).	☐	☐	☐	☐	☐	☐

14.a. If you want to, please add a few words to explain why this is your opinion.

15. How strongly do you agree or disagree with the following statement?

Please don't select more than 1 answer(s) per row.

	Strongly disagree	Disagree	Weakly disagree	Weakly agree	Agree	Strongly agree

The Medusa compatibility section should include details on where information came from e.g. references	☐	☐	☐	☐	☐	☐

15.a. If you want to, please add a few words to explain why this is your opinion.

Page 10: Any other information for the Medusa compatibility section

This is the last page! Please answer these two questions and then **click the 'Finish' button** below to save your previous answers.

16. Is there anything that has not been mentioned earlier in the survey that you think should be included in the Medusa compatibility section? If so, please describe it below.

17. How long have you been qualified (in years)?

Please enter a number.

Page 11: Thank you

Thank you for your responses, which will be very useful for improving Medusa.

Medusa Delphi survey - round 2

Page 1: Introduction

This is the **second of up to three related surveys** that you will be invited to complete about your opinions on the information on IV drug compatibility that should be provided in Medusa, the NHS Injectable Medicines Guide. **Your responses to all three surveys are important** and your contribution of valuable time to do this is much appreciated.

This survey is shorter than the first survey and will take about 15 minutes to complete. It repeats some of the questions from the first survey but **shows you an anonymous summary of the previous responses**, to find out if this changes people's views. In this way, it may be possible to develop agreement about what information on IV drug compatibility should be provided in Medusa.

This service evaluation project has been organised by the team that manages Medusa and is registered at Imperial College Healthcare NHS Trust (number 863). Only the Medusa team will have access to the information that you provide and it will not be shared with anyone else in an identifiable way. They will be treated as confidential and kept password protected. Once the three surveys are complete, the data will be anonymised and kept for at least 10 years. A summary of all responses may be published in reports about this project, but this will not include any identifiable information.

You are free to stop completing the survey at any time, without giving a reason. After you have clicked the finish button, it may not be possible to withdraw your data, as it will soon be used to prepare the next survey.

What do I do now?
If you would like to complete this survey, please answer the question below and click the 'Next' button. Please note that **the survey is not saved automatically**. Please answer as many questions as you want to and then move to the last page and **click the finish button**, otherwise your answers will not be saved.

If you wish to save your responses and finish the survey later, please click on the 'Finish later' option at the bottom of each page. This will take you to a new page with a link you can save to come back to your partly completed survey.

Thank you for your help.

1. If you have read the information above and agree to take part with the understanding that the data (including any personal data) you submit will be processed accordingly, please select the box below to get started. ✱ *Required*

○ I agree to take part

1 / 28

Page 2: Drugs for which compatibility information is provided

The information on this page has not changed from the first survey, apart from an extra explanation in the paragraph above the first image.

This survey is about the information provided by Medusa to support staff administering IV drugs via the same IV line or lumen. By this we mean administering:

- Two or more IV drugs
- At the same time
- Via the same IV line or lumen, so they mix before entering the bloodstream
- This usually requires use of a 'Y-site' connector, such as a double- or triple -lumen needle-free extension set (e.g. Octopus) or a ‘three way tap’.

This survey usually uses the word **'compatibility'** to mean information on whether two drugs are **stable or not** when mixed in the same IV line or lumen.

The next two questions are about the drug combinations for which compatibility information should be given, in order to provide a balance betwen comprehensiveness and information overload. You can think of this in two stages:

1. Which drugs should have compatibility information included in their IV guide on Medusa? This survey refers to these drugs as the '**primary drug**'. *For example, you might think it's important to provide this information for all drugs, or you might think it's only needed for certain drugs or those given by continuous infusion, etc*.
2. Once it has been decided to include compatibility information in the Medusa guide for a particular primary drug, we then need to decide which drug combinations with '**secondary drugs**' should be included. *For example, you might think it's important to provide all the available compatibility information, or you might think that information is only needed for compatibility with secondary drugs given by continuous or short infusion, etc.*

'Primary' and 'secondary' drugs are nothing to do with "clinical importance" - they simply describe whether the drug is the the main subject of the Medusa guide (primary drug), or another compatible drug refered to in that guide (secondary drug). The difference between primary and secondary drugs is illustrated in this screenshot from the Medusa monograph for fentanyl:

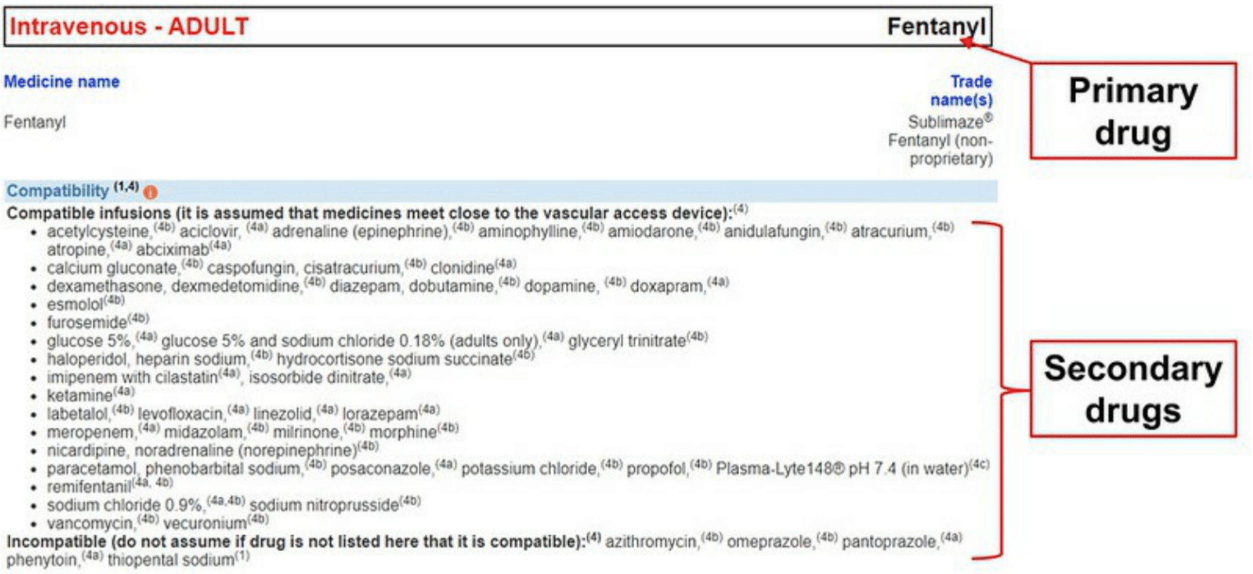

Intravenous - ADULT **Fentanyl**

Medicine name

Fentanyl

Trade name(s)

Sublimaze®
Fentanyl (non-proprietary)

Compatibility (1,4)

Compatible infusions (it is assumed that medicines meet close to the vascular access device):(4)

- acetylcysteine,(4b) aciclovir, (4a) adrenaline (epinephrine),(4b) aminophylline,(4b) amiodarone,(4b) anidulafungin,(4b) atracurium,(4b) atropine,(4a) abciximab(4a)
- calcium gluconate,(4b) caspofungin, cisatracurium,(4b) clonidine(4a)
- dexamethasone, dexmedetomidine,(4b) diazepam, dobutamine,(4b) dopamine, (4b) doxapram,(4a)
- esmolol(4b)
- furosemide(4b)
- glucose 5%,(4a) glucose 5% and sodium chloride 0.18% (adults only),(4a) glyceryl trinitrate(4b)
- haloperidol, heparin sodium,(4b) hydrocortisone sodium succinate(4b)
- imipenem with cilastatin(4a), isosorbide dinitrate,(4a)
- ketamine(4a)
- labetalol,(4b) levofloxacin,(4a) linezolid,(4a) lorazepam(4a)
- meropenem,(4a) midazolam,(4b) milrinone,(4b) morphine(4b)
- nicardipine, noradrenaline (norepinephrine)(4b)
- paracetamol, phenobarbital sodium,(4b) posaconazole,(4a) potassium chloride,(4b) propofol,(4b) Plasma-Lyte148® pH 7.4 (in water)(4c)
- remifentanil(4a, 4b)
- sodium chloride 0.9%,(4a,4b) sodium nitroprusside(4b)
- vancomycin,(4b) vecuronium(4b)

Incompatible (do not assume if drug is not listed here that it is compatible):(4) azithromycin,(4b) omeprazole,(4b) pantoprazole,(4a) phenytoin,(4a) thiopental sodium(1)

However, when considered from the point of view of the Medusa guide for adrenaline (now the primary drug), fentanyl becomes one of the secondary drugs:

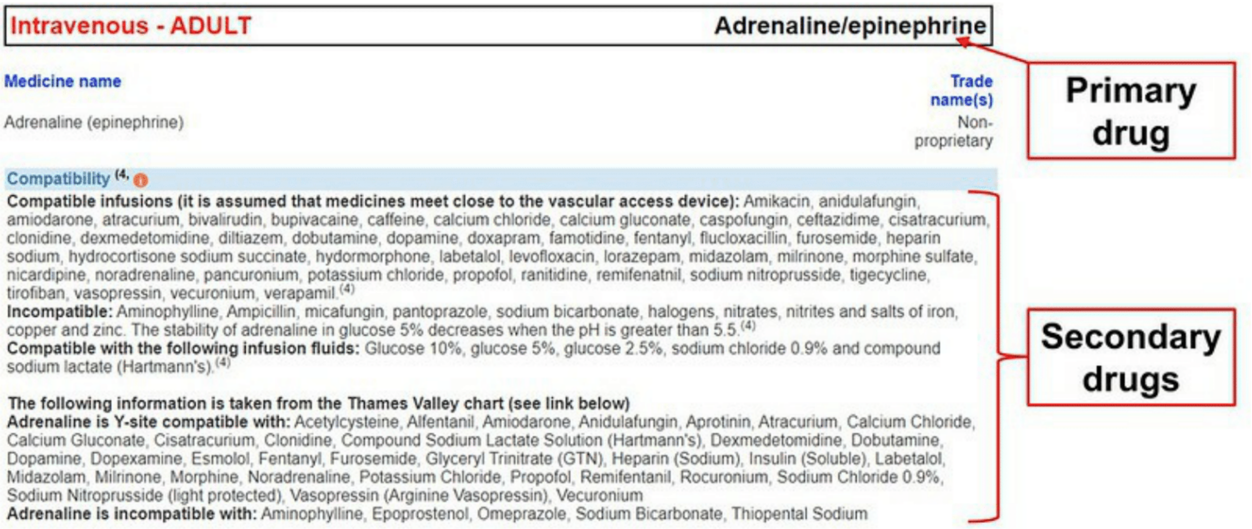

Intravenous - ADULT **Adrenaline/epinephrine**

Medicine name

Adrenaline (epinephrine)

Trade name(s)

Non-proprietary

Compatibility (4,

Compatible infusions (it is assumed that medicines meet close to the vascular access device): Amikacin, anidulafungin, amiodarone, atracurium, bivalirudin, bupivacaine, caffeine, calcium chloride, calcium gluconate, caspofungin, ceftazidime, cisatracurium, clonidine, dexmedetomidine, diltiazem, dobutamine, dopamine, doxapram, famotidine, fentanyl, flucloxacillin, furosemide, heparin sodium, hydrocortisone sodium succinate, hydormorphone, labetalol, levofloxacin, lorazepam, midazolam, milrinone, morphine sulfate, nicardipine, noradrenaline, pancuronium, potassium chloride, propofol, ranitidine, remifenatnil, sodium nitroprusside, tigecycline, tirofiban, vasopressin, vecuronium, verapamil.(4)
Incompatible: Aminophylline, Ampicillin, micafungin, pantoprazole, sodium bicarbonate, halogens, nitrates, nitrites and salts of iron, copper and zinc. The stability of adrenaline in glucose 5% decreases when the pH is greater than 5.5.(4)
Compatible with the following infusion fluids: Glucose 10%, glucose 5%, glucose 2.5%, sodium chloride 0.9% and compound sodium lactate (Hartmann's).(4)

The following information is taken from the Thames Valley chart (see link below)
Adrenaline is Y-site compatible with: Acetylcysteine, Alfentanil, Amiodarone, Anidulafungin, Aprotinin, Atracurium, Calcium Chloride, Calcium Gluconate, Cisatracurium, Clonidine, Compound Sodium Lactate Solution (Hartmann's), Dexmedetomidine, Dobutamine, Dopamine, Dopexamine, Esmolol, Fentanyl, Furosemide, Glyceryl Trinitrate (GTN), Heparin (Sodium), Insulin (Soluble), Labetalol, Midazolam, Milrinone, Morphine, Noradrenaline, Potassium Chloride, Propofol, Remifentanil, Rocuronium, Sodium Chloride 0.9%, Sodium Nitroprusside (light protected), Vasopressin (Arginine Vasopressin), Vecuronium
Adrenaline is incompatible with: Aminophylline, Epoprostenol, Omeprazole, Sodium Bicarbonate, Thiopental Sodium

The next page will ask about your opinions on the primary drugs that should have a compatibility section in Medusa.

Remember to answer as many questions as you want to and then move to the last page and **click the finish button**.

Page 3: Drugs for which compatibility information is provided

Before answering the questions below, please look at the percentages showing how participants responded in the first survey and then consider your own answer for this survey.

You may also want to consider these representative quotes from written responses to the first survey:

> "The shorter the time the dose of drug the less likely they will need to be put through the same access. Usually a cannula will be free or you could pause the infusion for a quick injection. The focus should remain on continuous infusions"
>
> "It is important to provide compatiblity information no matter how the intravenous drug is being infused."

2. How strongly do you agree or disagree with the following statement about the choice of the **primary drugs** for which compatibility information is provided? *The percentages show the responses given by all participants in the first survey.*

Please don't select more than 1 answer(s) per row.

	Strongly disagree (0%)	Disagree (3%)	Weakly disagree (0%)	Weakly agree (3%)	Agree (29%)	Strongly agree (65%)
All IV drug guides on Medusa for individual primary drugs given by CONTINUOUS INFUSION should include information on compatibility	☐	☐	☐	☐	☐	☐

3. How strongly do you agree or disagree with the following statement about the choice of the **primary drugs** for which compatibility information is provided? *The percentages show the responses given by all participants in the first survey.*

Please don't select more than 1 answer(s) per row.

	Strongly disagree (0%)	Disagree (6%)	Weakly disagree (6%)	Weakly agree (6%)	Agree (47%)	Strongly agree (35%)
All IV drug guides on Medusa for individual primary drugs given by SHORT INFUSION should include information on compatibility	☐	☐	☐	☐	☐	☐

4. How strongly do you agree or disagree with the following statement about the choice of the **primary drugs** for which compatibility information is provided? *The percentages show the responses given by all participants in the first survey.*

Please don't select more than 1 answer(s) per row.

	Strongly disagree (9%)	Disagree (20%)	Weakly disagree (17%)	Weakly agree (17%)	Agree (23%)	Strongly agree (14%)
All IV drug guides on Medusa for individual primary drugs given by INJECTION should include information on compatibility	☐	☐	☐	☐	☐	☐

5. If you want to, please add a few words to explain why this is your opinion.

The next page will ask about your opinions on the secondary drugs to include in the compatibility section of Medusa for selected primary drugs.

Remember to answer as many questions as you want to and then move to the last page and **click the finish button**

Page 4: Drugs for which compatibility information is provided

Before answering the questions below, please look at the percentages showing how participants responded in the first survey and then consider your own answer for this survey.

6. How strongly do you agree or disagree with the following statement about the choice of **secondary drugs** to include in the compatibility section of Medusa for selected primary drugs? *The percentages show the responses given by all participants in the first survey.*

Please don't select more than 1 answer(s) per row.

	Strongly disagree (0%)	Disagree (3%)	Weakly disagree (0%)	Weakly agree (0%)	Agree (41%)	Strongly agree (56%)
All secondary drugs given by CONTINUOUS INFUSION should be included	☐	☐	☐	☐	☐	☐

7. How strongly do you agree or disagree with the following statement about the choice of **secondary drugs** to include in the compatibility section of Medusa for selected primary drugs? *The percentages show the responses given by all participants in the first survey.*

Please don't select more than 1 answer(s) per row.

	Strongly disagree (0%)	Disagree (6%)	Weakly disagree (0%)	Weakly agree (6%)	Agree (62%)	Strongly agree (27%)
All secondary drugs given by SHORT INFUSION should be included	☐	☐	☐	☐	☐	☐

8. How strongly do you agree or disagree with the following statement about the choice of **secondary drugs** to include in the compatibility section of Medusa for selected primary drugs? *The percentages show the responses given by all participants in the first survey.*

Please don't select more than 1 answer(s) per row.

	Strongly disagree (3%)	Disagree (17%)	Weakly disagree (17%)	Weakly agree (26%)	Agree (20%)	Strongly agree (17%)

All secondary drugs given by INJECTION should be included	☐	☐	☐	☐	☐	☐

9. If you want to, please add a few words to explain why this is your opinion.

Page 5: Information in the Medusa compatibility section

After agreeing the drug combinations that should be included (i.e. the previous two questions), it's important to decide what information about these combinations should be provided and how strong the supporting data need to be. Once again, a balance betwen comprehensiveness and information overload is needed. The next two questions are about this issue.

Remember to answer as many questions as you want to and then move to the last page and **click the finish button**, otherwise your answers will not be saved.

Before answering the question below, please look at the percentages showing how participants responded in the first survey and then consider your own answer for this survey.

You may also want to consider these representative quotes from written responses to the first survey:

> "The implication of it being left off of the list implies the compatibility is not known. I think the only drugs where having a not known compatibility section would be those where you would expect there to be compatibility such as metaraminol."

> "The list of drugs is never going to be comprehensive. It may be confusing to see the difference between compatibility not know, and drug not listed."

10. How strongly do you agree or disagree with the following statement? *The percentages show the responses given by all participants in the first survey.*

Please don't select more than 1 answer(s) per row.

	Strongly disagree (9%)	Disagree (29%)	Weakly disagree (14%)	Weakly agree (6%)	Agree (20%)	Strongly agree (23%)
The Medusa compatibility section should list relevant drug combinations where compatibility is NOT KNOWN	☐	☐	☐	☐	☐	☐

11. If you want to, please add a few words to explain why this is your opinion.

Page 6: Information in the Medusa compatibility section

Before answering the questions below, please look at the percentages showing how participants responded in the first survey and then consider your own answer for this survey.

You may also want to consider these representative quotes from written responses to the first survey:

> "Incompatibilities which result in visible particles will always be chemically incompatible so there is a higher degree of certainty. Visual inspection may fail to detect a reaction which impacts on the concentration of medicine delivered incurring a risk to efficacy of the medicine."

> "Evidence of compatibility based on visual observation is weak and may not be accurate or consistent. However, if a combination has been observed to be incompatible, then this could be quoted."

12. How strongly do you agree or disagree with the following statement? *The percentages show the responses given by all participants in the first survey.*

Please don't select more than 1 answer(s) per row.

	Strongly disagree (3%)	Disagree (21%)	Weakly disagree (15%)	Weakly agree (18%)	Agree (38%)	Strongly agree (6%)
The Medusa compatibility section should state that drug combinations are COMPATIBLE based on reports of visual inspection of the mixture for signs of incompatibility	☐	☐	☐	☐	☐	☐

13. How strongly do you agree or disagree with the following statement? *The percentages show the responses given by all participants in the first survey.*

Please don't select more than 1 answer(s) per row.

	Strongly disagree (0%)	Disagree (12%)	Weakly disagree (9%)	Weakly agree (15%)	Agree (38%)	Strongly agree (27%)
The Medusa compatibility section should state that drug combinations are COMPATIBLE based on data from scientific instruments that detect particles	☐	☐	☐	☐	☐	☐

14. How strongly do you agree or disagree with the following statement? *The percentages show the responses given by all participants in the first survey.*

Please don't select more than 1 answer(s) per row.

	Strongly disagree (0%)	Disagree (6%)	Weakly disagree (3%)	Weakly agree (15%)	Agree (38%)	Strongly agree (38%)
The Medusa compatibility section should state that drug combinations are INCOMPATIBLE based on data from scientific instruments that detect particles	☐	☐	☐	☐	☐	☐

15. How strongly do you agree or disagree with the following statement? *The percentages show the responses given by all participants in the first survey.*

Please don't select more than 1 answer(s) per row.

	Strongly disagree (3%)	Disagree (9%)	Weakly disagree (9%)	Weakly agree (9%)	Agree (40%)	Strongly agree (31%)
The Medusa compatibility section should state that drug combinations are COMPATIBLE based on chemical analysis that can detect degradation of drugs that does NOT result in cloudiness or particle formation	☐	☐	☐	☐	☐	☐

16. How strongly do you agree or disagree with the following statement? *The percentages show the responses given by all participants in the first survey.*

Please don't select more than 1 answer(s) per row.

	Strongly disagree (0%)	Disagree (6%)	Weakly disagree (6%)	Weakly agree (9%)	Agree (47%)	Strongly agree (32%)

The Medusa compatibility section should state that drug combinations are INCOMPATIBLE based on chemical analysis that can detect degradation of drugs that does NOT result in cloudiness or particle formation	☐	☐	☐	☐	☐	☐

17. If you want to, please add a few words to explain why this is your opinion.

Page 7: Drug concentration information in the Medusa compatibility section

Finally, it's important to decide what additional details should be provided about drug combinations. Of course, a balance between comprehensiveness and information overload is needed. The rest of the survey is about this issue.

Remember to answer as many questions as you want to and then move to the last page and **click the finish button**, otherwise your answers will not be saved.

Before answering the questions below, please look at the percentages showing how participants responded in the first survey and then consider your own answer for this survey.

You may also want to consider these representative quotes from written responses to the first survey:

> "This helps make the compatibility relevant to practice and the situations in which they may be used."
>
> "Data should be as complete as possible to allow clinicians to make informed choices."
>
> "I think adding concentrations could cause confusion."
>
> "I think adding concentration information adds to information overload."

18. How strongly do you agree or disagree with the following statement? *The percentages show the responses given by all participants in the first survey.*

Please don't select more than 1 answer(s) per row.

	Strongly disagree (3%)	Disagree (11%)	Weakly disagree (6%)	Weakly agree (23%)	Agree (43%)	Strongly agree (14%)
The Medusa compatibility section should only state that a pair of drugs are compatible when the supporting data relate to drug concentrations used in practice	☐	☐	☐	☐	☐	☐

19. How strongly do you agree or disagree with the following statement? *The percentages show the responses given by all participants in the first survey.*

Please don't select more than 1 answer(s) per row.

	Strongly disagree (3%)	Disagree (9%)	Weakly disagree (3%)	Weakly agree (20%)	Agree (37%)	Strongly agree (29%)
The Medusa compatibility section should state the concentrations at which two drugs are compatible	☐	☐	☐	☐	☐	☐

20. If you want to, please add a few words to explain why this is your opinion.

Page 8: Infusion solution information in the Medusa compatibility section

Before answering the questions below, please look at the percentages showing how participants responded in the first survey and then consider your own answer for this survey.

You may also want to consider these representative quotes from written responses to the first survey:

> "Fluid selection is essential when dealing with patient with high sodium or blood glucose so we would need to know which fluids they were tested in."
>
> "Some medicines are incompatible in some diluents, and therefore should not be co-infused if the medicines are incompatible to the carrier solution of the other medication. This is a fairly uncommon situation but there are notable examples (eg amiodarone)."
>
> "It should only flag when one of the drugs is incompatible with one of the diluents."

21. How strongly do you agree or disagree with the following statement? *The percentages show the responses given by all participants in the first survey.*

Please don't select more than 1 answer(s) per row.

	Strongly disagree (0%)	Disagree (12%)	Weakly disagree (9%)	Weakly agree (18%)	Agree (38%)	Strongly agree (24%)
The Medusa compatibility section should only state that a pair of drugs are compatible when the supporting data relate to infusion solutions (diluents) used in practice	☐	☐	☐	☐	☐	☐

22. How strongly do you agree or disagree with the following statement? *The percentages show the responses given by all participants in the first survey.*

Please don't select more than 1 answer(s) per row.

	Strongly disagree (3%)	Disagree (6%)	Weakly disagree (3%)	Weakly agree (11%)	Agree (40%)	Strongly agree (37%)

The Medusa compatibility section should state the infusion solutions (diluents) in which two drugs are compatible	☐	☐	☐	☐	☐	☐

23. If you want to, please add a few words to explain why this is your opinion.

Page 9: Time scale information in the Medusa compatibility section

You're over halfway through the survey. Please keep going as your answers are important to the project.

Remember to answer as many questions as you want to and then move to the last page and **click the finish button**, otherwise your answers will not be saved.

Before answering the questions below, please look at the percentages showing how participants responded in the first survey and then consider your own answer for this survey.

You may also want to consider these representative quotes from written responses to the first survey:

> "This is useful context. If flow rates are very low, contact time is high. Again I would only do this if relevant (fairly uncommon)."
>
> "If there is a known incompatibility after a time period this is very important."
>
> "Most medications would be past the point of connection and into the patient before incompatibility from time issues become relevant."
>
> "This could be potentially confusing information. Medusa should only state two drugs are compatible if that is for a reasonable time frame relevant to practice."

24. How strongly do you agree or disagree with the following statement? *The percentages show the responses given by all participants in the first survey.*

Please don't select more than 1 answer(s) per row.

	Strongly disagree (0%)	Disagree (9%)	Weakly disagree (15%)	Weakly agree (24%)	Agree (41%)	Strongly agree (12%)
The Medusa compatibility section should only state that a pair of drugs are compatible when this is over a time scale relevant to practice	☐	☐	☐	☐	☐	☐

25. How strongly do you agree or disagree with the following statement? *The percentages show the responses given by all participants in the first survey.*

Please don't select more than 1 answer(s) per row.

	Strongly disagree (3%)	Disagree (9%)	Weakly disagree (11%)	Weakly agree (23%)	Agree (43%)	Strongly agree (11%)

The Medusa compatibility section should state for how long a pair of drugs are COMPATIBLE	☐	☐	☐	☐	☐	☐

26. How strongly do you agree or disagree with the following statement? *The percentages show the responses given by all participants in the first survey.*

Please don't select more than 1 answer(s) per row.

	Strongly disagree (0%)	Disagree (18%)	Weakly disagree (3%)	Weakly agree (27%)	Agree (27%)	Strongly agree (27%)
The Medusa compatibility section should state how long it takes a pair of drugs to become INCOMPATIBLE	☐	☐	☐	☐	☐	☐

27. If you want to, please add a few words to explain why this is your opinion.

Page 10: Temperature information in the Medusa compatibility section

Before answering the questions below, please look at the percentages showing how participants responded in the first survey and then consider your own answer for this survey.

You may also want to consider these representative quotes from written responses to the first survey:

> "Temparature isn't something we can change easily, the compatibility chart should be based on standard room temperature."
>
> "Not sure how relevant this information would be in practice."
>
> "I find increasing temperature monitoring would be confusing for staff members and result in lots of infusions being stopped if small deviations of temperature occur."
>
> "The evidence may be at certain temperatures but may not matter so useful to know to make clinical judgement."

28. How strongly do you agree or disagree with the following statement? *The percentages show the responses given by all participants in the first survey.*

Please don't select more than 1 answer(s) per row.

	Strongly disagree (0%)	Disagree (15%)	Weakly disagree (3%)	Weakly agree (35%)	Agree (44%)	Strongly agree (3%)
The Medusa compatibility section should only state that a pair of drugs are compatible when the supporting data relate to temperatures relevant to administration (not storage) in practice	☐	☐	☐	☐	☐	☐

29. How strongly do you agree or disagree with the following statement? *The percentages show the responses given by all participants in the first survey.*

Please don't select more than 1 answer(s) per row.

	Strongly disagree (9%)	Disagree (17%)	Weakly disagree (3%)	Weakly agree (34%)	Agree (29%)	Strongly agree (9%)

The Medusa compatibility section should state the temperatures at which two drugs are compatible	☐	☐	☐	☐	☐	☐

30. If you want to, please add a few words to explain why this is your opinion.

Page 11: Protection from light information in the Medusa compatibility section

Before answering the questions below, please look at the percentages showing how participants responded in the first survey and then consider your own answer for this survey.

You may also want to consider these representative quotes from written responses to the first survey:

> "A protect from light statement should be included where relevant but doesn't need to be in every monograph"

> "If it is not stated whether light is a consideration I would want to assume that the products were not kept in a dark environment."

31. How strongly do you agree or disagree with the following statement? *The percentages show the responses given by all participants in the first survey.*

Please don't select more than 1 answer(s) per row.

	Strongly disagree (3%)	Disagree (18%)	Weakly disagree (24%)	Weakly agree (24%)	Agree (21%)	Strongly agree (12%)
The Medusa compatibility section should only state that a pair of drugs are compatible when the supporting data relate to administration in light (not protected from light)	☐	☐	☐	☐	☐	☐

32. How strongly do you agree or disagree with the following statement? *This is a revised question, so there are no results from the first survey.*

Please don't select more than 1 answer(s) per row.

	Strongly disagree	Disagree	Weakly disagree	Weakly agree	Agree	Strongly agree
The Medusa compatibility section should state when two drugs are compatible in light	☐	☐	☐	☐	☐	☐

33. How strongly do you agree or disagree with the following statement? *This is a revised question, so*

there are no results from the first survey.

Please don't select more than 1 answer(s) per row.

	Strongly disagree	Disagree	Weakly disagree	Weakly agree	Agree	Strongly agree
The Medusa compatibility section should state when two drugs are only compatible in darkness	☐	☐	☐	☐	☐	☐

34. If you want to, please add a few words to explain why this is your opinion.

Page 12: Material information in the Medusa compatibility section

You're nearly at the end of the survey. Please keep going as your answers are important to the project.

Remember to answer as many questions as you want to and then move to the last page and **click the finish button**, otherwise your answers will not be saved.

Before answering the questions below, please look at the percentages showing how participants responded in the first survey and then consider your own answer for this survey.

You may also want to consider these representative quotes from written responses to the first survey:

> "Important if specific line composition is required."
>
> "If this is known to affect the outcome then it should be included."
>
> "I think it's hard for front-line clinical staff to know what the containers are made out of. So is this information overload? Monograph writers on the other hand may have more time to consider this information."

35. How strongly do you agree or disagree with the following statement? *The percentages show the responses given by all participants in the first survey.*

Please don't select more than 1 answer(s) per row.

	Strongly disagree (6%)	Disagree (20%)	Weakly disagree (17%)	Weakly agree (23%)	Agree (20%)	Strongly agree (14%)
The Medusa compatibility section should only state that a pair of drugs are compatible when the supporting data relate to an IV container and/or IV line material used in practice	☐	☐	☐	☐	☐	☐

36. How strongly do you agree or disagree with the following statement? *The percentages show the responses given by all participants in the first survey.*

Please don't select more than 1 answer(s) per row.

	Strongly disagree (0%)	Disagree (15%)	Weakly disagree (6%)	Weakly agree (12%)	Agree (41%)	Strongly agree (27%)

If there are specific requirements, the Medusa compatibility section should state what the IV container and/or IV line containing a mixture of two drugs should be made from (e.g. glass, PVC)	☐	☐	☐	☐	☐	☐

37. If you want to, please add a few words to explain why this is your opinion.

Page 13: Mixing information in the Medusa compatibility section

Before answering the question below, please look at the percentages showing how participants responded in the first survey and then consider your own answer for this survey.

You may also want to consider these representative quotes from written responses to the first survey:

> "If compatible mixing in same container will also be compatible during Y-site administration."

> "If compatibility data is available for mixing in a container is available then it would be useful to state it."

38. How strongly do you agree or disagree with the following statement? *The percentages show the responses given by all participants in the first survey.*

Please don't select more than 1 answer(s) per row.

	Strongly disagree (9%)	Disagree (14%)	Weakly disagree (6%)	Weakly agree (9%)	Agree (43%)	Strongly agree (20%)
The Medusa compatibility section should state that a pair of drugs are compatible when the supporting data relate to mixing in the same container (e.g. bag, syringe)	☐	☐	☐	☐	☐	☐

39. If you want to, please add a few words to explain why this is your opinion.

Page 14: Additional information in the Medusa compatibility section

This is the last page! Please answer these three questions and then **click the 'Finish' button** below to save your previous answers.

Before answering the questions below, please look at the percentages showing how participants responded in the first survey and then consider your own answer for this survey.

You may also want to consider these representative quotes from written responses to the first survey:

> "Irrelevant for clinical staff, and adds to bulk of information."

> "Allows for risk assessment for individual patients with complex needs."

40. How strongly do you agree or disagree with the following statement? *The percentages show the responses given by all participants in the first survey.*

Please don't select more than 1 answer(s) per row.

	Strongly disagree (3%)	Disagree (9%)	Weakly disagree (14%)	Weakly agree (20%)	Agree (29%)	Strongly agree (26%)
The Medusa compatibility section should include information on how the compatibility data on which it is based was obtained, e.g. visual inspection for cloudiness, chemical analysis etc.	☐	☐	☐	☐	☐	☐

40.a. If you want to, please add a few words to explain why this is your opinion.

You may also want to consider these representative quotes from written responses to the first survey:

> "This will help in the case of unknown compatibility and help inform a risk assessment."

> "Repeating information from other sections could increase the length of the overall monograph and reduce readability."

> "Essential for national extravasation strategy."

> "Not required in this section as data is already presented in another format."

41. How strongly do you agree or disagree with the following statement? *The percentages show the responses given by all participants in the first survey.*

Please don't select more than 1 answer(s) per row.

	Strongly disagree (9%)	Disagree (18%)	Weakly disagree (18%)	Weakly agree (21%)	Agree (21%)	Strongly agree (15%)
The Medusa compatibility section should include the pH of the individual drugs in ADDITION to it already being provided in the separate pH section.	☐	☐	☐	☐	☐	☐

41.a. If you want to, please add a few words to explain why this is your opinion.

You may also want to consider these representative quotes from written responses to the first survey:

> "Adds to bulk of information and leads to confusion. That information should be held elsewhere if someone has a query related to this, but focus on simple, uncluttered, easy to read monographs for front line staff to read in busy, noisy clinical areas."
>
> "Not sure I would chase up the references. Would probably just trust what Medusa says."
>
> "Medusa maintains a reputation for high quality because of the meticulous attention to detail including provision of references for all information."
>
> "This helps users such as Pharmacy understand what data has already been considered so reducing duplication of effort."

42. How strongly do you agree or disagree with the following statement? *The percentages show the responses given by all participants in the first survey.*

Please don't select more than 1 answer(s) per row.

	Strongly disagree (3%)	Disagree (3%)	Weakly disagree (12%)	Weakly agree (18%)	Agree (29%)	Strongly agree (35%)
The Medusa compatibility section should include details on where information came from e.g. references	☐	☐	☐	☐	☐	☐

42.a. If you want to, please add a few words to explain why this is your opinion.

Page 15: Thank you

Thank you for your responses, which will be very useful for improving Medusa.

Medusa Delphi survey - round 3

Page 1: Introduction

This is the last **of three related surveys** that you will be invited to complete about your opinions on the information on IV drug compatibility that should be provided in Medusa, the NHS Injectable Medicines Guide. **Your responses to all three surveys are important** and your contribution of valuable time to do this is much appreciated.

This survey is shorter than the first two surveys and will take 10-15 minutes to complete. It repeats some of the questions from the first two surveys but **shows you an anonymous summary of the previous responses**, to find out if this changes people's views. In this way, it may be possible to develop agreement about what information on IV drug compatibility should be provided in Medusa.

This service evaluation project has been organised by the team that manages Medusa and is registered at Imperial College Healthcare NHS Trust (number 863). Only the Medusa team will have access to the information that you provide and it will not be shared with anyone else in an identifiable way. They will be treated as confidential and kept password protected. Once the three surveys are complete, the data will be anonymised and kept for at least 10 years. A summary of all responses may be published in reports about this project, but this will not include any identifiable information.

You are free to stop completing the survey at any time, without giving a reason. After you have clicked the finish button, it may not be possible to withdraw your data, as it will soon be used to prepare the next survey.

What do I do now?
If you would like to complete this survey, please answer the question below and click the 'Next' button. Please note that **the survey is not saved automatically**. Please answer as many questions as you want to and then move to the last page and **click the finish button**, otherwise your answers will not be saved.

If you wish to save your responses and finish the survey later, please click on the 'Finish later' option at the bottom of each page. This will take you to a new page with a link you can save to come back to your partly completed survey.

Thank you for your help.

1. If you have read the information above and agree to take part with the understanding that the data (including any personal data) you submit will be processed accordingly, please select the box below to get started. ✱ *Required*

○ I agree to take part

Page 2: Drugs for which compatibility information is provided

This survey is about the information provided by Medusa to support staff administering IV drugs via the same IV line or lumen. By this we mean administering:

- Two or more IV drugs
- At the same time
- Via the same IV line or lumen, so they mix before entering the bloodstream
- This usually requires use of a 'Y-site' connector, such as a double- or triple -lumen needle-free extension set (e.g. Octopus) or a ‘three way tap’.

This survey usually uses the word **'compatibility'** to mean information on whether two drugs are **stable or not** when mixed in the same IV line or lumen.

The two questions on the next page are about the drug combinations for which compatibility information should be given, in order to provide a balance betwen comprehensiveness and information overload. You can think of this in two stages:

1. Which drugs should have compatibility information included in their IV guide on Medusa?
2. Once it has been decided to include compatibility information in the Medusa guide for a particular drug, we then need to decide which drug combinations should be included.

Remember to answer as many questions as you want to and then move to the last page and **click the finish button**.

Page 3: Drugs for which compatibility information is provided

Before answering the questions below, please look at the percentages showing how participants responded in the second survey and then consider your own answer for this survey.

You may also want to consider these representative quotes from written responses to the first survey:

> "Continuous infusions are likely to need to be run along side something else. A bolus is highly unlikely to need to be given with anything else (or at least the risk can be mitigated e.g. pause other infusions etc)."
>
> "As long as adequate flushing occurs pre and post bolus, continuous infusions can be interrupted to administer an injection through the same venous access."
>
> "With limited IV access (e.g. 1 cannula) it is useful to know whether I would need to stop maintenance fluids (or continuous drug infusion) to give bolus doses or short infusion of a drug."
>
> "It is important to know the compatibility of medication no matter of the duration of administration."

2. How strongly do you agree or disagree with the following statement? *The percentages show the responses given by all participants in the second survey.*

Please don't select more than 1 answer(s) per row.

	Strongly disagree (3%)	Disagree (17%)	Weakly disagree (14%)	Weakly agree (40%)	Agree (6%)	Strongly agree (20%)

All IV guides on Medusa for drugs given by INJECTION ONLY should include information on their compatibility with other drugs	☐	☐	☐	☐	☐	☐

3. How strongly do you agree or disagree with the following statement? *The percentages show the responses given by all participants in the second survey.*

Please don't select more than 1 answer(s) per row.

	Strongly disagree (3%)	Disagree (20%)	Weakly disagree (11%)	Weakly agree (34%)	Agree (17%)	Strongly agree (14%)
All IV guides on Medusa for drugs given by CONTINUOUS OR SHORT INFUSION should include information on their compatibility with drugs given by INJECTION ONLY	☐	☐	☐	☐	☐	☐

4. If you want to, please add a few words to explain why this is your opinion.

Remember to answer as many questions as you want to and then move to the last page and **click the finish button**

Page 4: Information in the Medusa compatibility section

After agreeing the drug combinations that should be included (i.e. the previous two questions), it's important to decide what information about these combinations should be provided and how strong the supporting data need to be. Once again, a balance betwen comprehensiveness and information overload is needed. The next two questions are about this issue.

Remember to answer as many questions as you want to and then move to the last page and **click the finish button**, otherwise your answers will not be saved.

Before answering the question below, please look at the percentages showing how participants responded in the second survey and then consider your own answer for this survey.

You may also want to consider these representative quotes from written responses to the second survey:

> "It acknowledges that there is no known info, which means I don't have to go and look for data if that's a combo I'm interested in."
>
> "Drug combinations where compatibility are not known should not be listed to prevent confusion and the absence of information should not require inclusion."
>
> "It doesn't add anything and would be difficult to provide a comprehensive list."

5. How strongly do you agree or disagree with the following statement? *The percentages show the responses given by all participants in the second survey.*

Please don't select more than 1 answer(s) per row.

	Strongly disagree (11%)	Disagree (31%)	Weakly disagree (14%)	Weakly agree (11%)	Agree (20%)	Strongly agree (11%)

7 / 27

The Medusa compatibility section should list COMMON, IMPORTANT drug combinations where compatibility is NOT KNOWN	☐	☐	☐	☐	☐	☐

5.a. If you want to, please add a few words to explain why this is your opinion.

6. How strongly do you agree or disagree with the following statement? *This is a new question, so there are no percentages or quotations to look at.*

Please don't select more than 1 answer(s) per row.

	Strongly disagree	Disagree	Weakly disagree	Weakly agree	Agree	Strongly agree

In ADDITION to 'Y-site' compatibility information, the Medusa compatibility section should include information on the mixing of drugs in the SAME CONTAINER (e.g. infusion bag, syringe)	☐	☐	☐	☐	☐	☐

6.a. If you want to, please add a few words to explain why this is your opinion.

Page 5: Information in the Medusa compatibility section

Before answering the questions below, please look at the percentages showing how participants responded in the second survey and then consider your own answer for this survey.

You may also want to consider these representative quotes from written responses to the second survey:

> "Visual inspection should be considered in absence of data from testing. For compatible drugs maybe it could flag that there is weak evidence of compatibility in these cases."

> "Incompatibility should be listed whether found as a result of chemical analysis or particle formation."

> "Known incompatibility of drugs based on chemical analysis that do not result in visual changes would be the most valuable information. If we do not know compatibility status but have no other option than to combine two drugs then all we have available is visual changes to the products. Most compatibility data I view on resources other than Medusa only has visual changes listed however, so I don't want it to make it appear that accepted practice of drug combinations has been incorrect due to lack of chemical analytical studies."

7. How strongly do you agree or disagree with the following statement? *The percentages show the responses given by all participants in the second survey.*

Please don't select more than 1 answer(s) per row.

	Strongly disagree (6%)	Disagree (29%)	Weakly disagree (9%)	Weakly agree (14%)	Agree (40%)	Strongly agree (3%)

10 / 27

The Medusa compatibility section should state that drug combinations are COMPATIBLE based on reports of visual inspection of the mixture for signs of incompatibility	☐	☐	☐	☐	☐	☐

8. How strongly do you agree or disagree with the following statement? *The percentages show the responses given by all participants in the second survey.*

Please don't select more than 1 answer(s) per row.

	Strongly disagree (3%)	Disagree (6%)	Weakly disagree (3%)	Weakly agree (9%)	Agree (54%)	Strongly agree (26%)
The Medusa compatibility section should state that drug combinations are COMPATIBLE based on data from scientific instruments that detect particles	☐	☐	☐	☐	☐	☐

9. How strongly do you agree or disagree with the following statement? *The percentages show the responses given by all participants in the second survey.*

Please don't select more than 1 answer(s) per row.

	Strongly disagree (3%)	Disagree (3%)	Weakly disagree (0%)	Weakly agree (3%)	Agree (49%)	Strongly agree (43%)
The Medusa compatibility section should state that drug combinations are INCOMPATIBLE based on data from scientific instruments that detect particles	☐	☐	☐	☐	☐	☐

10. If you want to, please add a few words to explain why this is your opinion.

Page 6: Drug concentration information in the Medusa compatibility section

Finally, it's important to decide what additional details should be provided about drug combinations. Of course, a balance between comprehensiveness and information overload is needed. The rest of the survey is about this issue.

Remember to answer as many questions as you want to and then move to the last page and **click the finish button**, otherwise your answers will not be saved.

Before answering the questions below, please look at the percentages showing how participants responded in the second survey and then consider your own answer for this survey.

You may also want to consider these representative quotes from written responses to the second survey:

> "Having additional information helps when at times we have to administer drug concentrations beyond the usual practice."
>
> "The information is subjective without this."
>
> "I think listing the maximum concentrations where a two drug combination has been stable will be most valuable. If there is variable compatibility depending on concentration the maximum and minimum should be listed. When we have fluid restricted concentrations of drugs we can't assume these are as compatible with other drugs as their standard dilution counterparts."
>
> "There may be a level of extrapolation required but should not include when concentrations are very different. Should be assumed that concentrations refer to the range recommended in the monograph. Only would consider referring to concentration if two very different methods of administration e.g. neat injection against infusion diluted."

11. How strongly do you agree or disagree with the following statement? *The percentages show the responses given by all participants in the second survey.*

Please don't select more than 1 answer(s) per row.

	Strongly disagree (6%)	Disagree (6%)	Weakly disagree (3%)	Weakly agree (20%)	Agree (46%)	Strongly agree (20%)
The Medusa compatibility section should only state that a pair of drugs are compatible when the supporting data relate to drug concentrations used in practice	☐	☐	☐	☐	☐	☐

12. How strongly do you agree or disagree with the following statement? *The percentages show the responses given by all participants in the second survey.*

Please don't select more than 1 answer(s) per row.

	Strongly disagree (0%)	Disagree (3%)	Weakly disagree (0%)	Weakly agree (26%)	Agree (43%)	Strongly agree (29%)
The Medusa compatibility section should state the concentrations at which two drugs are compatible	☐	☐	☐	☐	☐	☐

13. If you want to, please add a few words to explain why this is your opinion.

Page 7: Infusion solution information in the Medusa compatibility section

Before answering the question below, please look at the percentages showing how participants responded in the second survey and then consider your own answer for this survey.

14. How strongly do you agree or disagree with the following statement? *The percentages show the responses given by all participants in the second survey.*

Please don't select more than 1 answer(s) per row.

	Strongly disagree (0%)	Disagree (6%)	Weakly disagree (9%)	Weakly agree (6%)	Agree (54%)	Strongly agree (26%)
The Medusa compatibility section should only state that a pair of drugs are compatible when the supporting data relate to infusion solutions (diluents) used in practice	☐	☐	☐	☐	☐	☐

14.a. If you want to, please add a few words to explain why this is your opinion.

Page 8: Time scale information in the Medusa compatibility section

You're over halfway through the survey. Please keep going as your answers are important to the project.

Remember to answer as many questions as you want to and then move to the last page and **click the finish button**, otherwise your answers will not be saved.

Before answering the questions below, please look at the percentages showing how participants responded in the second survey and then consider your own answer for this survey.

You may also want to consider these representative quotes from written responses to the second survey:

> "If there is a known incompatibility after a time period this is very important."
>
> "I'm the most interested in whether there is a known timescale by which point a combination becomes incompatible."

15. How strongly do you agree or disagree with the following statement? *The percentages show the responses given by all participants in the second survey.*

Please don't select more than 1 answer(s) per row.

	Strongly disagree (3%)	Disagree (9%)	Weakly disagree (6%)	Weakly agree (23%)	Agree (43%)	Strongly agree (17%)
The Medusa compatibility section should state for how long a pair of drugs are COMPATIBLE	☐	☐	☐	☐	☐	☐

16. How strongly do you agree or disagree with the following statement? *The percentages show the responses given by all participants in the second survey.*

Please don't select more than 1 answer(s) per row.

	Strongly disagree (3%)	Disagree (20%)	Weakly disagree (0%)	Weakly agree (11%)	Agree (31%)	Strongly agree (34%)
The Medusa compatibility section should state how long it takes a pair of drugs to become INCOMPATIBLE	☐	☐	☐	☐	☐	☐

17. If you want to, please add a few words to explain why this is your opinion.

Page 9: Temperature information in the Medusa compatibility section

Before answering the question below, please look at the percentages showing how participants responded in the second survey and then consider your own answer for this survey.

You may also want to consider these representative quotes from written responses to the second survey:

> "Fridge and room temp are very different things and compatibility is likely to have been tested at one or the other or both so worth stating when known."

> "Important in the case of prolonged cardiac surgery where body temperature and room temperature are greatly reduced."

18. How strongly do you agree or disagree with the following statement? *The percentages show the responses given by all participants in the second survey.*

Please don't select more than 1 answer(s) per row.

	Strongly disagree (3%)	Disagree (14%)	Weakly disagree (9%)	Weakly agree (49%)	Agree (20%)	Strongly agree (6%)
The Medusa compatibility section should state the temperatures at which two drugs are compatible	☐	☐	☐	☐	☐	☐

18.a. If you want to, please add a few words to explain why this is your opinion.

18 / 27

Page 10: Protection from light information in the Medusa compatibility section

You're nearly at the end of the survey. Please keep going as your answers are important to the project.

Remember to answer as many questions as you want to and then move to the last page and **click the finish button**, otherwise your answers will not be saved.

Before answering the questions below, please look at the percentages showing how participants responded in the second survey and then consider your own answer for this survey.

You may also want to consider these representative quotes from written responses to the second survey:

> "Administration in light and darkness are terms unfamiliar in clinical practice."

> "I think it is assumed that a pair of drugs are compatible in light unless Medusa says that they are not."

19. How strongly do you agree or disagree with the following statement? *The percentages show the responses given by all participants in the second survey.*

Please don't select more than 1 answer(s) per row.

	Strongly disagree (3%)	Disagree (12%)	Weakly disagree (24%)	Weakly agree (29%)	Agree (27%)	Strongly agree (6%)

The Medusa compatibility section should only state that a pair of drugs are compatible when the supporting data relate to administration in light (not protected from light)	☐	☐	☐	☐	☐	☐

20. How strongly do you agree or disagree with the following statement? *The percentages show the responses given by all participants in the second survey.*

Please don't select more than 1 answer(s) per row.

	Strongly disagree (6%)	Disagree (11%)	Weakly disagree (23%)	Weakly agree (23%)	Agree (31%)	Strongly agree (6%)
The Medusa compatibility section should state when two drugs are compatible in light	☐	☐	☐	☐	☐	☐

21. How strongly do you agree or disagree with the following statement? *The percentages show the responses given by all participants in the second survey.*

Please don't select more than 1 answer(s) per row.

	Strongly disagree (0%)	Disagree (17%)	Weakly disagree (14%)	Weakly agree (17%)	Agree (23%)	Strongly agree (29%)

The Medusa compatibility section should state when two drugs are only compatible in darkness	☐	☐	☐	☐	☐	☐

22. If you want to, please add a few words to explain why this is your opinion.

Page 11: Material information in the Medusa compatibility section

Before answering the question below, please look at the percentages showing how participants responded in the second survey and then consider your own answer for this survey.

23. How strongly do you agree or disagree with the following statement? *The percentages show the responses given by all participants in the second survey.*

Please don't select more than 1 answer(s) per row.

	Strongly disagree (0%)	Disagree (17%)	Weakly disagree (9%)	Weakly agree (17%)	Agree (43%)	Strongly agree (14%)
The Medusa compatibility section should only state that a pair of drugs are compatible when the supporting data relate to an IV container and/or IV line material used in practice	☐	☐	☐	☐	☐	☐

23.a. If you want to, please add a few words to explain why this is your opinion.

Page 12: Additional information in the Medusa compatibility section

This is the last page! Please answer these three questions and then **click the 'Finish' button** below to save your previous answers.

Before answering the questions below, please look at the percentages showing how participants responded in the second survey and then consider your own answer for this survey.

You may also want to consider these representative quotes from written responses to the second survey:

> "Helps to identify how robust the data is and inform any needed clinical risk assessment."
>
> "If that much detail is needed should refer to original source."

24. How strongly do you agree or disagree with the following statement? *The percentages show the responses given by all participants in the second survey.*

Please don't select more than 1 answer(s) per row.

	Strongly disagree (0%)	Disagree (3%)	Weakly disagree (9%)	Weakly agree (23%)	Agree (26%)	Strongly agree (40%)

The Medusa compatibility section should include information on how the compatibility data on which it is based was obtained, e.g. visual inspection for cloudiness, chemical analysis etc.	☐	☐	☐	☐	☐	☐

24.a. If you want to, please add a few words to explain why this is your opinion.

You may also want to consider these representative quotes from written responses to the second survey:

"Can be looked up easily enough elsewhere in the monographs."

"Duplication. This information is already included in detail in the monograph."

"This has been useful especially deciding what access does patient need."

25. How strongly do you agree or disagree with the following statement? *The percentages show the responses given by all participants in the second survey.*

Please don't select more than 1 answer(s) per row.

	Strongly disagree (9%)	Disagree (26%)	Weakly disagree (14%)	Weakly agree (31%)	Agree (9%)	Strongly agree (11%)

The Medusa compatibility section should include the pH of the individual drugs in ADDITION to it already being provided in the separate pH section.	☐	☐	☐	☐	☐	☐

25.a. If you want to, please add a few words to explain why this is your opinion.

You may also want to consider these representative quotes from written responses to the second survey:

> "I would want to appraise where information may have come from and consider any resources that may not have been used. "
>
> "While I recognise this can lead to a lot of info on the page, this can be in small font at the bottom so that it isn't distracting. We need to know the references so that we know the information is correct and of high quality."

26. How strongly do you agree or disagree with the following statement? *The percentages show the responses given by all participants in the second survey.*

Please don't select more than 1 answer(s) per row.

	Strongly disagree (6%)	Disagree (9%)	Weakly disagree (11%)	Weakly agree (20%)	Agree (17%)	Strongly agree (37%)

The Medusa compatibility section should include details on where information came from e.g. references	☐	☐	☐	☐	☐	☐

26.a. If you want to, please add a few words to explain why this is your opinion.

27. How long have you been qualified (in years)?

Page 13: Thank you

Thank you for your responses, which will be very useful for improving Medusa.
