# Supplementary material for: Evaluation of NHS Injectable Medicines Guide users’ information needs related to the co-infusion of intravenous medicines: user survey and Delphi consensus study
Source: BMJ Open. 2025 May 30;15(5):e094211. doi: 10.1136/bmjopen-2024-094211 (PMC12128442; doi:10.1136/bmjopen-2024-094211)
Supplement: Supplementary file 2 [file bmjopen-2024-094211.reviewer_comments.pdf]

PEER REVIEW HISTORY

BMJ Open publishes all reviews undertaken for accepted manuscripts. Reviewers are asked to complete a checklist review form and are provided with free text boxes to elaborate on their assessment. These free text comments are reproduced below.

ARTICLE DETAILS

Title (Provisional)

Evaluation of NHS Injectable Medicines Guide users' information needs related to the co-infusion of intravenous medicines via a user survey and Delphi consensus study

Authors

Jones, Matthew; Aguado Lorenzo, Virginia

VERSION 1 - REVIEW

**Reviewer**	**1**
**Name**	**Paiva-Santos, Filipe**
**Affiliation**	**Higher School of Nursing of Coimbra Health Sciences Research Unit Nursing, Fundamental Nursing**
**Date**	**11-Dec-2024**
**COI**	**None**

Well-written manuscript supported by current and relevant references.

The introduction is adequate, focused on the problem.

The methods are clear and in line with the objectives. Although it is mentioned that the participants consented to participate in the study, there is no reference to the approval of an ethics committee, and I am sure it is an accidental omission.

Results well presented and in line with the method.

The discussion is poor, making little confrontation with literature. Although the authors point out that the number of participants below what was initially intended is a limitation, I ask why they did not decide to extend the data collection period? The recommendations listed are pertinent and relevant.

The conclusion responds to the objectives clearly.

**Reviewer**	**2**
**Name**	**Christensen, Robin**

Affiliation **Copenhagen University Hospital, Frederiksberg, The Parker Institute: MSU**

Date **21-Mar-2025**

COI **I am among the statistical advisors to the journal (BMJ Open)**

As a statistical advisor to the journal, I have been asked to assess the robustness of the methodology and reporting. Having been involved in several Delphi projects (many subsequently published in respected journals), I reviewed the manuscript with great interest.

The authors present a study aimed at gathering insights from healthcare professionals and (other) experts to develop consensus recommendations on the intravenous drug compatibility information that should be included in 'Medusa' to enhance clinical practice and patient safety.

Q1: First and foremost, it is essential for the authors to remember that the journal requires all proportions to be reported alongside the absolute count (e.g., XX [yy%]);

e.g., abstract currently states: “68% of users were involved in the……”

Q2: From the abstract it was difficult to see whether the ‘Main Analysis’ was based on stake-holder individuals who completed all three rounds of Delphi?

Q3: While the methodology and corresponding reporting are commendable and set a high standard, I encourage the authors to develop a more concise and informative "Results" section, particularly in the Abstract. Without this, the valuable survey work may not effectively resonate with the reader.

- Ad Q3: Based on my understanding of the authors' evidence synthesis (summary), the following suggestions may be considered: specifying which estimands (with confidence intervals, if possi-ble) are needed to support the objectives that appear to justify this study.

The co-infusion of drugs is common (XX [yy%, 95%CI: xy to yz%]), so supporting information continues to be important. The most relevant information is that identified by both users (XX [yy%] of survey respondents) and the expert panel (XX [yy%] agreement).

- Maybe list these core areas (all stakeholders agree) explicitly with some inferential measures (say if >70% agree?).

Q4: In summary, clear, and explicit objectives are essential for guiding both the design and analysis of a study. As the authors are already aware of, well-defined “objectives” represent testable (and falsifiable) hypotheses, providing a solid foundation for selecting appropriate sta-tistical methods and estimands. If need be, I encourage the authors to reconsider their original objectives, as doing so would allow for explicit reporting of results, typically with

estimates and 95% confidence intervals, thereby enhancing transparency and interpretability.

While the methodology and reporting are commendable and set a high standard, I sincerely en-courage the authors to refine the objectives—perhaps even post hoc—to make them more con-crete and measurable. This will significantly enhance the clarity and impact of the study.

VERSION 1 - AUTHOR RESPONSE

Reviewer: 1

Dr. Filipe Paiva-Santos, Higher School of Nursing of Coimbra Health Sciences Research Unit Nursing

Comment 1.1: Although it is mentioned that the participants consented to participate in the study, there is no reference to the approval of an ethics committee, and I am sure it is an accidental omission.

Response 1.1: Under the UK Policy Framework for Health and Social Care Research this project was categorised as a service evaluation of Medusa by Imperial College Healthcare NHS Trust, therefore review by a research ethics committee was not required. However, the project applied the same ethical standards as would be expected by an ethics committee, considering aspects such as participant wellbeing, informed consent and confidentiality. The design and project materials were therefore reviewed and approved by the Pharmacy Department Quality and Safety Committee and the Clinical Audit Team of Imperial College Healthcare NHS Trust on 4th May, 2023 (number 863). We have revised the text in the second paragraph of the Methods section to make this clearer.

Comment 1.2: The discussion is poor, making little confrontation with literature.

Response 1.2: Thank you for this comment. However, after careful consideration of the relevant literature, we believe that this is well discussed (as described below), especially given the small number of previous studies in this area.

Paragraph 3 of the discussion links to previous research on the frequency of IV medicine co-infusion and the commonly co-infused drugs. It also highlights the tension between a previous UK safety alert and the research literature, including the findings of this study. The 'Recommendations' sub-section also engages with the literature, including that relating to frequently co-infused drug combinations, and the best ways to present compatibility information. It also draws on previous research to recommend user testing of revised designs for the Medusa compatibility section. In response to this comment,

we have slightly expanded this by adding details of two previous studies that linked difficulties finding and understanding information in Medusa with medication errors ('Recommendations' sub-section, paragraph 2).

Comment 1.3: Although the authors point out that the number of participants below what was initially intended is a limitation, I ask why they did not decide to extend the data collection period?

Response 1.3: The survey was closed in September 2023 as the rate of recruitment at this point was very slow and all potential routes for advertising the study had been used. We therefore decided there was little chance of recruiting significantly more participants. This has been clarified in the 'User survey: participants and recruitment' sub-section of the 'Methods' section.

Reviewer: 2
Dr. Robin Christensen, Frederiksberg and Bispebjerg Hospital

Comment 2.1: First and foremost, it is essential for the authors to remember that the journal requires all proportions to be reported alongside the absolute count (e.g., XX [yy%]); e.g., abstract currently states: "68% of users were involved in the……"

Response 2.1: Thank you for highlighting this. We have corrected this wherever percentages are used to present the findings – in the abstract, first paragraph of the 'Results' section, and Tables 2 and 3.

Comment 2.2: From the abstract it was difficult to see whether the 'Main Analysis' was based on stake-holder individuals who completed all three rounds of Delphi?

Response 2.2: Analysis was based on all responses submitted to each round, not only those from participants who completed all three rounds. This is partly because the Delphi method requires analysis to take place between rounds, when it is not known which participants will subsequently complete all the rounds. We have clarified this in paragraph 1 of the 'Delphi project' sub-section of the 'Results' section.

Comment 2.3: While the methodology and corresponding reporting are commendable and set a high standard, I encourage the authors to develop a more concise and informative "Results" section, particularly in the Abstract. Without this, the valuable survey work may not effectively resonate with the reader.
- Ad Q3: Based on my understanding of the authors' evidence synthesis (summary), the following suggestions may be considered: specifying which estimands (with confidence

intervals, if possible) are needed to support the objectives that appear to justify this study.
The co-infusion of drugs is common (XX [yy%, 95%CI: xy to yz%]), so supporting information continues to be important. The most relevant information is that identified by both users (XX [yy%] of survey respondents) and the expert panel (XX [yy%] agreement).
- Maybe list these core areas (all stakeholders agree) explicitly with some inferential measures (say if >70% agree?).

Response 2.3: We'd like to thank Dr. Christensen for his kind comment on our methodology and reporting standards. We have revised both the abstract and the 'Results' section. In the abstract, we have more numerical data, including confidence intervals for proportions, inferential test results and the pre-specified definitions of agreement for the Delphi study. As suggested, we have also listed the core areas of agreement in the conclusion of the abstract. In the main 'Results' section, we have added 95% confidence intervals wherever relevant and linked specific results to a numbered objective.

Comment 2.4: In summary, clear, and explicit objectives are essential for guiding both the design and analysis of a study. As the authors are already aware of, well-defined "objectives" represent testable (and falsifiable) hypotheses, providing a solid foundation for selecting appropriate statistical methods and estimands. If need be, I encourage the authors to reconsider their original objectives, as doing so would allow for explicit reporting of results, typically with estimates and 95% confidence intervals, thereby enhancing transparency and interpretability.
While the methodology and reporting are commendable and set a high standard, I sincerely encourage the authors to refine the objectives—perhaps even post hoc—to make them more concrete and measurable. This will significantly enhance the clarity and impact of the study.

Response 2.4: thank you for this suggestion. We have refined the objectives (at the end of the 'Introduction' section) to restate our original intentions in a more granular way. We have then referred to objective numbers in the 'Methods', 'Results' and 'Discussion' sections to clarify the links between certain questions/results and specific objectives. As stated above, we have also added 95% confidence intervals wherever relevant.

VERSION 2 - REVIEW

**Reviewer**	**2**
**Name**	**Christensen, Robin**

Affiliation **Copenhagen University Hospital, Frederiksberg, The Parker Institute: MSU**

Date **06-May-2025**

COI

I think the authors did a fair job addressing my statistical queries, although the stated objective — particularly in the abstract — is still not directly applicable. Since I'm not sure how I could improve it myself, I'm inclined to accept this latest version.
